# Emission Dynamics of Constitutive and Herbivore-induced Plant Volatiles from Norway Maple (*Acer platanoides*) Trunk Infested by the Asian Longhorned Beetle (*Anoplophora glabripennis* (Motschulsky))

**DOI:** 10.1007/s10886-026-01718-2

**Published:** 2026-05-21

**Authors:** Jennifer Braun, Robin Tost, Markus Witzler, Carsten Engelhard, Peter Kaul

**Affiliations:** 1 Institute of Safety and Security Research, Bonn-Rhein-Sieg University of Applied Sciences, Von-Liebig-Str. 20, 53359 Rheinbach, Germany; 2 Department of Chemistry and Biology and Research Center of Micro- and Nanochemistry and (Bio)Technology (Cµ), University of Siegen, Adolf-Reichwein-Str. 2, 57068 Siegen, Germany; 3 Bundesanstalt Für Materialforschung Und -Prüfung (BAM), Richard-Willstätter-Str. 11, 12489 Berlin, Germany

**Keywords:** *Anoplophora glabripennis* (Motschulsky), *Acer platanoides*, Invasive pest, Constitutive trunk-volatiles, Herbivore-induced plant volatiles, TD–GC–MS

## Abstract

Early detection of the Asian longhorned beetle (*Anoplophora glabripennis*, ALB), a destructive wood-boring pest, remains challenging in living trees. Volatile organic compounds (VOCs) have gained increasing attention as potential detection cues due to their key roles in plant–insect interactions, yet trunk-based volatile responses to wood-boring herbivores are still poorly understood. Here, we present a detailed case study characterizing constitutive and herbivore-induced plant volatiles (HIPVs) emitted from a living *Acer platanoides* trunk infested by ALB. Over six months, trunk emissions were monitored and analyzed noninvasively using thermal desorption gas chromatography mass spectrometry (TD–GC–MS). Constitutive emissions were dominated by aldehydes and were primarily influenced by abiotic factors such as temperature and light, whereas ALB infestation had only minor effects on their emission rates. In contrast, HIPVs were characterized mainly by terpenoids and exhibited infestation-stage–specific dynamics: monoterpenoids – dominated by p-cymene, (*E*)-4,8-dimethylnona-1,3,7-triene, and γ-terpinene – prevailed during oviposition, whereas sesquiterpenoids dominated during larval feeding, particularly cyclosativene, α-longipinene, α-copaene, zingiberene, and α-curcumene. In addition, nitrogen- and oxygen-containing volatiles were identified as previously unreported components of the ALB-induced trunk volatile blend. Several induced terpenoids are known to elicit electrophysiological or behavioral responses in ALB and its natural enemies, and therefore represent valuable key compounds that can be utilized for olfactory detection purposes. Though requiring validation with replications and analogous experiments with further plant and insect species, the findings of this study provide valuable mechanistic insights in tritrophic interactions between plant, wood-boring insect and its enemies.

**Supplementary Information:**

The online version contains supplementary material available at 10.1007/s10886-026-01718-2.

## Introduction

Integrated pest management (IPM) is becoming increasingly important in response to the growing number of invasive species and their impact on the local ecosystem. According to the Intergovernmental Science-Policy Platform on Biodiversity and Ecosystem Services (IPBES), invasive alien species are among the key drivers of biodiversity loss and ecosystem degradation, necessitating effective and sustainable management strategies (IPBES 2023). A central goal of IPM is to improve detection and control of pests through the integration of biological, chemical, and ecological knowledge, including the use of volatile organic compounds (VOCs) as diagnostic and functional tools (Smart et al. 2014).

Plants emit VOCs as part of their dynamic interaction with the environment (Dudareva et al. 2006, 2013). These compounds fulfill multiple ecological functions, including attraction of pollinators and seed disseminators (Raguso 2008), mitigation of abiotic stress such as excessive light, temperature, or drought (Vickers et al. 2009; Loreto and Schnitzler 2010), defense against herbivores (Unsicker et al. 2009) and pathogens (Kaur et al. 2022), and communication with neighboring plants (Baldwin et al. 2006). Among these, herbivore-induced plant volatiles (HIPVs) play a particularly important role in plant defense (Chen 2008; Aljbory and Chen 2018). HIPVs comprise diverse chemical classes, including terpenoids, fatty acid derivatives, phenylpropanoids or benzenoids, and sulphur- or nitrogen-containing compounds (Mumm and Dicke 2010; Aljbory and Chen 2018). The qualitative and quantitative composition of HIPV profiles is influenced by plant and herbivore species, developmental stages, and environmental conditions (Arimura et al. 2009). HIPVs can mediate direct defense by deterring herbivores through toxic, repellent, or anti-nutritive effects, and indirect defense by attracting predators and parasitoids that reduce the number of attacking herbivores (War et al. 2012). Furthermore, HIPVs can prime intact parts of the same plant or nearby plants, enhancing their resistance to future herbivory (Hu et al. 2018). Owing to these multifunctional roles, HIPVs offer considerable potential for application in IPM and pest monitoring strategies (Smart et al. 2014). One invasive pest that causes enormous damage worldwide is the Asian longhorned beetle (*Anoplophora glabripennis* Motschulsky, Coleoptera: Cerambycidae, ALB) (Wang et al. 2023). Native to East Asia, ALB is a highly destructive, polyphagous wood-boring pest targeting deciduous hardwoods of various plant families such as Sapindaceae (*Acer*, *Aesculus*), Betulaceae (*Betula*), Fagaceae (*Fagus*), Platanaceae (*Platanus*), Salicaceae (*Populus*, *Salix*), and Ulmaceae (*Ulmus*) (van der Gaag and Loomans 2014; Wang et al. 2023). Even within its native range, ALB has caused extensive damage by destroying several millions of trees, particularly in northern China (Wang et al. 2023). Introduced through solid wood packaging material (WPM), ALB has expanded its dispersion area to Europe and North America (Commission Implementing Regulation (EU) 2025/1952). Eradication programs have incurred costs of millions of US and Canadian dollars in the United States and Canada and hundreds of thousands of Euros in Europe (EPPO 2026). In response, the Commission on Phytosanitary Measures of the International Plant Protection Convention (IPPC) adopted the International Standard for Phytosanitary Measures No. 15 (ISPM 15) to “reduce the risk of introduction and spread of quarantine pests associated with the movement in international trade of wood packaging material made from raw wood” (FAO 2018). This regulated lowers WPM infestation rates by approximately one-third to one-half (Haack et al. 2014), but it also imposes economic costs of international trade; currently modest under the present ISPM 15 requirements, yet substantially higher under stricter implementation scenarios (Strutt et al. 2013).

The life cycle of the ALB begins with the female laying eggs beneath the bark into the cambium (Meng et al. 2015; Lyu et al. 2023). After hatching, larvae undergo five or more development stages (instars) and feed for 1–2 years on various parts of the tree trunk (Smith et al. 2002; Keena and Moore 2010), causing extensive internal damage that compromises both structural integrity and physiological function of the host tree. Following overwintering, pupation usually occurs in spring or summer (Keena and Moore 2010; Haack et al. 2018), and adults emerge within 1–2 weeks after eclosion (Sánchez and Keena 2013). While adults feed briefly on twigs and leaf tissues, larval feeding accounts for the majority of tree mortality and economic damage (Xu and Teale 2021). Since the ALB is classified as a priority Union quarantine pest across the European Union, its management is regulated by strict eradication measures, including removal of infested trees and potential hosts within a radius of 100 m, followed by intensive monitoring of buffer zones (2 km) for at least four years (Comission Implementing Regulation (EU) 2025/1952). Current monitoring techniques rely primarily on visual surveys for signs of infestation such as adult emergence holes, oviposition pits, larval frass, and feeding tunnels (Meng et al. 2015; Wang et al. 2023), suffering from low detection rates for standing trees (20–36%), being even lower for recent infestations (Eyre and Barbrook 2021). The use of mobile elevated platforms or tree climbers, pheromone traps, and detection dogs can improve detection, but remain limited in their capabilities. Pheromone traps, though effective for many flying insects, have not yet proven operationally effective for ALB (Xu and Teale 2021). Detection dogs have demonstrated high sensitivity (75–88%) and specificity (85–96%) for identifying larval frass in test scenarios (Hoyer-Tomiczek et al. 2016), but the availability and accessibility of training material is limited due to its quarantine status, and the dogs’ effectiveness in field conditions is often inconsistent (Arnesen and Rosell 2021). Consequently, there is a strong need for complementary, noninvasive detection approaches that enable earlier and more reliable identification of infested trees.

Considerable effort has been devoted to identifying ALB-related semiochemicals, including host kairomones emitted from leaves, branches, and flowers (Li et al. 2003; Zhang et al. 2008; Hu et al. 2009; Wang et al. 2016), as well as sex pheromones produced by adult beetles (Nehme et al. 2009; Wickham et al. 2012; Xu et al. 2020, 2021), both complemented by or combined with behavioral studies of adult ALB beetles (Zhang et al. 2008; Nehme et al. 2009, 2010; Scholz and Schütz 2012). Knowledge of attractants was utilized for developing pheromone traps targeting adult ALB beetles (Lyu et al. 2023), whereas understanding repellent semiochemicals aids in elucidating host selection preferences (Zhang et al. 2008) and in designing forests and parks with less attractive, more resistant tree species. Despite numerous studies on semiochemicals, HIPVs emitted from leaves and petioles upon feeding of adult beetles or from trunks following oviposition and larval feeding gained less attention. Although particularly relevant for wood-boring insects, trunks represent a relevant yet understudied source of HIPVs (Rissanen et al. 2020). Given that the ALB remains in the larval stage for most of its life cycle and that its natural enemies typically attack immature stages, trunk-emitted HIPVs may play an important ecological role in integrated pest management.

Natural enemies of the ALB include predators (*e.g.* woodpeckers), entomopathogens (*e.g.* fungi and bacteria), and parasitoids (*e.g.* beetles or wasps) (Wang et al. 2023). To date, 29 parasitoid species from two insect orders and seven families have been reported (Johnson et al. 2025), of which the beetle *Dastarcus helophoroides* (Fairmaire) (Coleoptera: Bothrideridae) (Wang et al. 2023) and hymenopteran wasps of the genus *Sclerodermus* (Hymenoptera: Bethylidae), are the most intensively studied and ecologically relevant (Men et al. 2019). Both are generalist ectoparasitoids that target different development stages of ALB. *D. helophoroides* parasitizes late-instar larvae, pupae, and young adults of several longhorned beetle species (Wei et al. 2008), whereas hymenopteran wasps primarily attack eggs or early-instar larvae (Li et al. 2020; Johnson et al. 2025).

Beyond their ecological function, trunk-emitted HIPVs may also provide valuable chemical markers for noninvasive detection of ALB infestations, particularly during periods when adult beetles are absent. Previous studies have identified several VOCs associated with ALB infestation including copaene, (+)-cyclosativene, (+)-α-longipinene, zingiberene, 2,4-dimethyl-1-heptene, and 3-carene (Makarow et al. 2019, 2020), with some confirmed as ALB-specific using multivariate analysis (Vermeeren et al. 2025). However, information on qualitative and quantitative trunk emission profiles, their temporal dynamics, and their potential roles in indirect defense remains largely lacking.

The aim of this detailed case study was to characterize constitutive and herbivore-induced volatile emissions from a living *Acer platanoides* trunk infested by ALB and to examine their temporal dynamics over a six-month period using noninvasive sampling and thermal desorption gas chromatography mass spectrometry (TD–GC–MS). Specifically, we sought to (i) elucidate constitutive trunk VOC profiles and their modulation by abiotic stress, (ii) identify HIPVs induced by ALB oviposition and larval feeding as well as by neighboring infestation, and (iii) evaluate the potential roles of these volatiles in indirect defense strategies and plant–plant signaling. This study aimed to advance understanding of tritrophic interactions by characterizing volatiles with particular importance in deterring further herbivores and attracting natural enemies. By leveraging nature’s defense strategies, these compounds offer promising signaling cues for developing targeted VOC-based tools for the early detection and monitoring of ALB infestations.

## Methods and Materials

### Plants and Insects

**Fig. 1:**
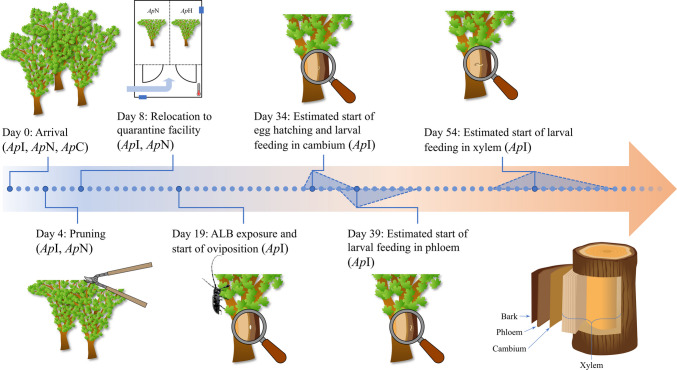
Timeline of treatments to Acer platanoides with ApC being the non-infested control outside the quarantine facility, ApN the non-infested neighbor inside the quarantine facility and ApI the ALB-infested tree. Developmental times of ALB stages and their uncertainties (visualized by dashed triangles) were estimated based on interpolation of literature data (Keena 2006; Keena and Moore 2010)

Three individuals of *Acer platanoides* (Sapindaceae) were provided by a local tree nursery where they had been cultivated outdoors. Trees were excavated, transported to the facilities of the Bonn-Rhein-Sieg University of Applied Sciences (H–BRS, Rheinbach, Germany), and kept in pots (230 L). One tree served as control, remained outside the quarantine facility and did not undergo further treatment (*Ap*C). The other two trees were given time to acclimate before they were pruned to a height of approximately 2 m and placed into separate chambers inside the quarantine facility (Fig. 1). The quarantine facility was a 26 m^2^ ventilated room and the two chambers were separated by metal grids (Fig. S1). Each chamber was equipped with Samsung LM561c FULL Spectrum 3000/3500/5000K-LED-MIX plant lamps (GrowArt Profitechnik für die Pflanzenzucht, Germany), providing full light spectrum for 12 h per day. One tree (*Ap*I) was infested with *Anoplophora glabripennis* (Motschulsky) (Coleoptera: Cerambycidae), while the neighboring tree (*Ap*N) remained noninfested (Fig. 1).

ALB larvae were provided by the University of Göttingen (Göttingen, Germany) and by the Julius Kühn-Institute (Braunschweig, Germany), where insects were reared on artificial diet. After adult eclosion, beetles were maintained individually in laboratory glass bottles and fed with fresh maple leaves until sexual maturity. Subsequently, two female and two male adults were released onto *Ap*I and remained on the tree for their entire life span. Copulation and oviposition pits were observed on the day of beetle release. As destructive sampling was avoided, larval developmental stages were estimated based on the temperature inside the quarantine facility (25 ± 3 °C) and literature data (Keena 2006; Keena and Moore 2010). At 25 °C, eggs were expected to start hatching approximately 2 weeks after oviposition, with first instar larvae start feeding in the cambium. By about 3 weeks, larvae were expected to start migrating to the phloem, and bored deeper into the xylem by 5 weeks after oviposition, where feeding continued (Fig. 1). Uncertainties resulting from temperature fluctuations in the range of 22–28 °C were estimated by fitting literature data using the ‘Asymptotic1’ function by OriginLab (Fig. S2). The treatment timeline to all *Ap* trees and estimated larval developmental stages including their uncertainties are visualized in Fig. 1.

### Sampling Procedure and Analysis

Trunk VOC emissions of *Ap*I and *Ap*N were sampled on 42 days over a 188-day period. *Ap*C was sampled on 34 days within the first 138 days due to *Ap*C’s declining vitality, possibly due to waterlogging as no signs of plant diseases were observed. Two consecutive samples were collected per tree per sampling day within the same time window to minimize diurnal variation. The total number of samples per tree is summarized in Table 1. For VOC collection, a 10–20 cm trunk section (~ 2 dm^2^ bark surface) was enclosed using Nalophan® foil, secured with staples and foam padded straps (adapted from Makarow et al*.*) (Makarow et al. 2019). The same trunk sections were sampled throughout the experiment. In order to differentiate trunk volatiles from ubiquitous volatiles, background measurements were taken from the surrounding air. VOCs were enriched on adsorbent tubes containing Tenax® TA at 30 mL min^−1^ for 90 min using a portable air pump (Gilian GilAir Plus, Sensidyne, LP, FL, USA). Adsorbent tubes were stored in sealed glass tubes with PTFE-lined caps at − 18 °C until analysis.

**Table 1:** Grouping of samples and total number of measurements (N) from *A. platanoides* trunks of non-infested control (*Ap*C), ALB-infested maple (*Ap*I), and non-infested neighbor (*Ap*N), and from background (BG) measurements outside and inside the quarantine facility (Q)

		*Acer platanoides*			Background	
Phase	Days	*Ap*C	*Ap*I	*Ap*N	Outside Q	Inside Q
(1) Acclimation	0–8	Healthy (N = 13)	Healthy (N = 13)	Healthy (N = 13)	Healthy (N = 5)	n.a
(2) Abiotic stress	9–19	Healthy (N = 7)	Stressed (N = 7)	Stressed (N = 7)	Healthy (N = 4)	Stressed (N = 4)
(3) Biotic stress	20–188	Healthy (N = 45)	Infested (N = 61)	Neighbored (N = 63)	Healthy (N = 20)	Infested (N = 30)

TD–GC–MS measurements were performed using a 7890B/5977B MSD GC–MS instrument (Agilent Technologies, CA, USA) coupled to a TD-unit from Gerstel (GERSTEL GmbH & Co.KG, Mülheim an der Ruhr, Germany). The GC was equipped with a HP-5MS capillary column (30 m × 0.25 mm × 0.25 µm, Agilent Technologies, CA, USA). Helium 5.0 (Westfalen AG, Münster, Germany) was used as carrier gas at a constant flow rate of 1.2 mL min^−1^. The analysis was performed according to the method developed by Makarow et al*.* (Makarow et al. 2019). Briefly, the TD was initially set to 30 °C, heated to 230 °C at 40 °C min^−1^ and held for 1 min. The desorbed volatiles were trapped in a cooled injection system (CIS) at − 120 °C, then heated to 250 °C at 12 °C min^−1^ and held for 3 min. The GC temperature program started at 35 °C for 2 min, increased to 170 °C at 8 °C min^−1^, then to 240 °C at 60 °C min^−1^, and was held at 240 °C for 2 min. The analysis was carried out with a 1:10 split, applied between CIS and GC. Mass spectra were recorded with a single quadrupole after electron impact (EI) ionization (70 eV) over the mass-to-charge (*m/z*) range of 35–400.

### Chemicals

Adsorbent tubes were filled with 80 mg Tenax® TA (60/80 mesh) (Buchem BV, Apeldoorn, Netherlands), fixed with silanized glass wool (Merck KGaA, Darmstadt, Germany). Nalophan® foil (Kalle GmbH, Wiesbaden, Germany) made of polyethylene terephthalate (PET) was used for enclosing the sampled tree trunks.

The certified reference materials (CRMs) SVOC Mixture 748 (1000 μg mL^−1^ in methanol), Terpene Mixtures 1 and 2 (100 µg mL^−1^ in methanol), Carbonyl Compounds Mixture 876 (1000 μg mL^−1^ in acetonitrile), and EPA Method 8260 VOC Mixture 565 (200 μg mL^−1^ in methanol) were purchased from LGC Standards GmbH (Wesel, Germany), and the Aliphatics Mix (C_5_–C_12_, 2000 µg mL^−1^ in methanol) from Merck KGaA (Darmstadt, Germany). The analytical standards 2-methylbutane (≥ 99%), 1-penten-3—ol (99%), 3-methylbutanal (97%), 2-methylbutanal (≥ 95%), p-cymene (99%), ocimene (mixture of isomers, ≥ 90%), (+)-α-longipinene (≥ 97%), and heptadecane (99%) were purchased from Merck KGaA (Darmstadt, Germany). Isobutyronitrile (> 95%), 1-octene (≥ 99%), 2-ethyl-1-hexanol (≥ 99%), α-santalene (> 95%), trans-α-bergamotene (> 95%), zingiberene (> 95%), and cuparene (> 95%) were purchased from LGC Standards GmbH (Wesel, Germany). 2,4-Dimethyl-1-heptene (98%) was purchased from Combi-Blocks (CA, USA), benzaldehyde (≥ 99%) from Th. Geyer GmbH & Co. KG (Renningen, Germany), benzyl alcohol (99%) from Fisher Scientific GmbH (Schwerte, Germany), and α-copaene (≥ 95%) from Cayman Chemicals (MI, USA). Analytical standards were dissolved and diluted in methanol (PESTINORM® Supra Trace for organic trace analysis, ≥ 99.9%, VWR Chemicals, Darmstadt, Germany).

### Compound Identification and Quantification

Data processing was performed Agilent MassHunter Workstation Quantitative Analysis – Unknowns Analysis Version 10.2 (Agilent Technologies, CA, USA). Peaks were detected by deconvolution and filtered by an absolute height threshold greater than 10^3^ counts. Tentative compound identification was based on comparison of deconvoluted mass spectra with the NIST 20 EI mass spectral library and verification by linear retention indices (LRI), calculated using *n*-alkane standards (*n*C_5_–*n*C_17_) according to van Den Dool and Kratz (van Den Dool and Kratz 1963). Where available, compound identities were confirmed with CRMs or authentic standards.

Emission rates were derived from component areas calculated via the deconvolution algorithm as the summed ion intensities associated with the deconvoluted component. Where applicable, component areas were converted to mass units through quantification with CRMs or authentic standards. Stock solutions of analytical standards were prepared in methanol (10 mg mL^−1^). Four to eight calibration levels, covering the relevant concentration range of each compound, were prepared by diluting CRMs or stock solutions of analytical standards in methanol. A 1 µL aliquot of each calibration solution was injected onto an adsorbent tube and dried by pumping clean air through the tubes at a flow rate of 300 mL min^−1^ for 3 min. Terpenoids without available authentic standards were tentatively quantified using structurally related terpenoids with similar retention behavior. Emission rates were normalized to sampling time and sampled bark area and expressed as either component area per square decimeter per hour (× 10^6^ dm^−2^ h^−1^) or nanograms per square decimeter per hour (ng dm^−2^ h^−1^).

### Data Processing and Statistical Analysis

The study was limited to one tree per treatment category by practical constraints of working with a quarantine pest in a spatially limited facility. To nonetheless ensure a reliability of detected compounds with sufficiently consistent occurrence, only those with a detection frequency (DF) ≥ 10% across all maple samples (N = 229) were further processed with OriginPro 2021b (v9.8.5.201, OriginLab Corporation, MA, USA). The experimental timeline was divided into three distinct periods (Table 1) to differentiate between constitutive emissions and those induced by abiotic (pruning and relocation) or biotic stress (direct or neighboring infestation). For statistical grouped analysis, consecutively taken samples on the same day were counted as two samples. In this study, constitutive emissions are defined as compounds that are continuously emitted rather than transiently expressed. Consequently, a DF threshold of 50% was applied to all trees prior to biotic stress treatment (periods 1 − 2) to identify these core volatiles. Kruskal–Wallis rank sum test, followed by Dunn’s post hoc test (*P* < 0.05) was then used to test significance of differences in emission rates of constitutive volatiles from differently treated trees within the same experimental phase (inter-tree, within a row in Table 1), and from the same tree between different phases (intra-tree, within a column in Table 1).

Plant volatiles induced by stress, direct or neighbored herbivory were identified at the individual trees. Compounds emitted by *Ap*I in period 3 were considered herbivore-induced (HIPV) if they appeared de novo after infestation or increased ≥ twofold in *Ap*I compared to both its pre-infestation phase and control trees (*Ap*N and *Ap*C). Similarly, compounds emitted by *Ap*N were considered induced in the neighboring tree if they appeared de novo or increased ≥ twofold in emission compared to *Ap*I and *Ap*C. Furthermore, compounds whose emission increased ≥ twofold and to a comparable extent in both *Ap*I and *Ap*N were considered stress-induced.

Relevant plant volatiles were analyzed for their temporal emission dynamics throughout the measurement period. For this purpose, emissions were averaged per day and visualized as means ± standard errors of means of double determinations (M ± SE). Where appropriate, temporal patterns with component areas or nanograms per square decimeter per hour ($y$) plotted against the time in days ($x$) were fitted using the ‘Extreme’ peak function by OriginLab. According to Eq. (1), ${x}_{c}$ denotes the position of the peak in days, ${y}_{0}$ the emission height offset, $A$ the peak amplitude, and $w$ denotes the peak width.

1 $$\begin{array}{c}y={y}_{0}+A{e}^{\left(-{e}^{\left(-z\right)}-z+1\right)}\\ with z=(x-{x}_{c})/w\end{array}$$

In addition, Kendall’s tau correlation coefficients (τ) were calculated to assess correlations in emission dynamics of induced volatiles within the same compound class.

## Results

Across the six-month monitoring period, more than 200 VOCs were detected from the trunks of Norway maples (*A. platanoides*), comprising both constitutive and induced emissions. 35 compounds were consistently detected in ≥ 50% of measurements of three individual trees prior to biotic stress and were classified as constitutive trunk volatiles. An additional 111 compounds were classified as induced volatiles, based on de novo occurrence or at least twofold increase in emission rates in intra- and inter-tree comparisons following abiotic stress, direct ALB herbivory, or neighboring herbivory.

Among the induced compounds, 46 were detected in ≥ 50% of measurements from at least one tree following stress treatment, and a further 44 occurred in ≥ 30% of measurements. Most induced volatiles (76%) were associated with the ALB-infested maple (*Ap*I), whereas 18% were induced in the neighboring maple (*Ap*N), and 7% increased comparably in both *Ap*I and *Ap*N relative to the noninfested control (*Ap*C). The following sections describe the temporal dynamics and chemical composition of constitutive and induced trunk volatiles, with emphasis on ALB herbivory-related responses.

### Temporal Dynamics of VOC Emissions

**Fig. 2:**
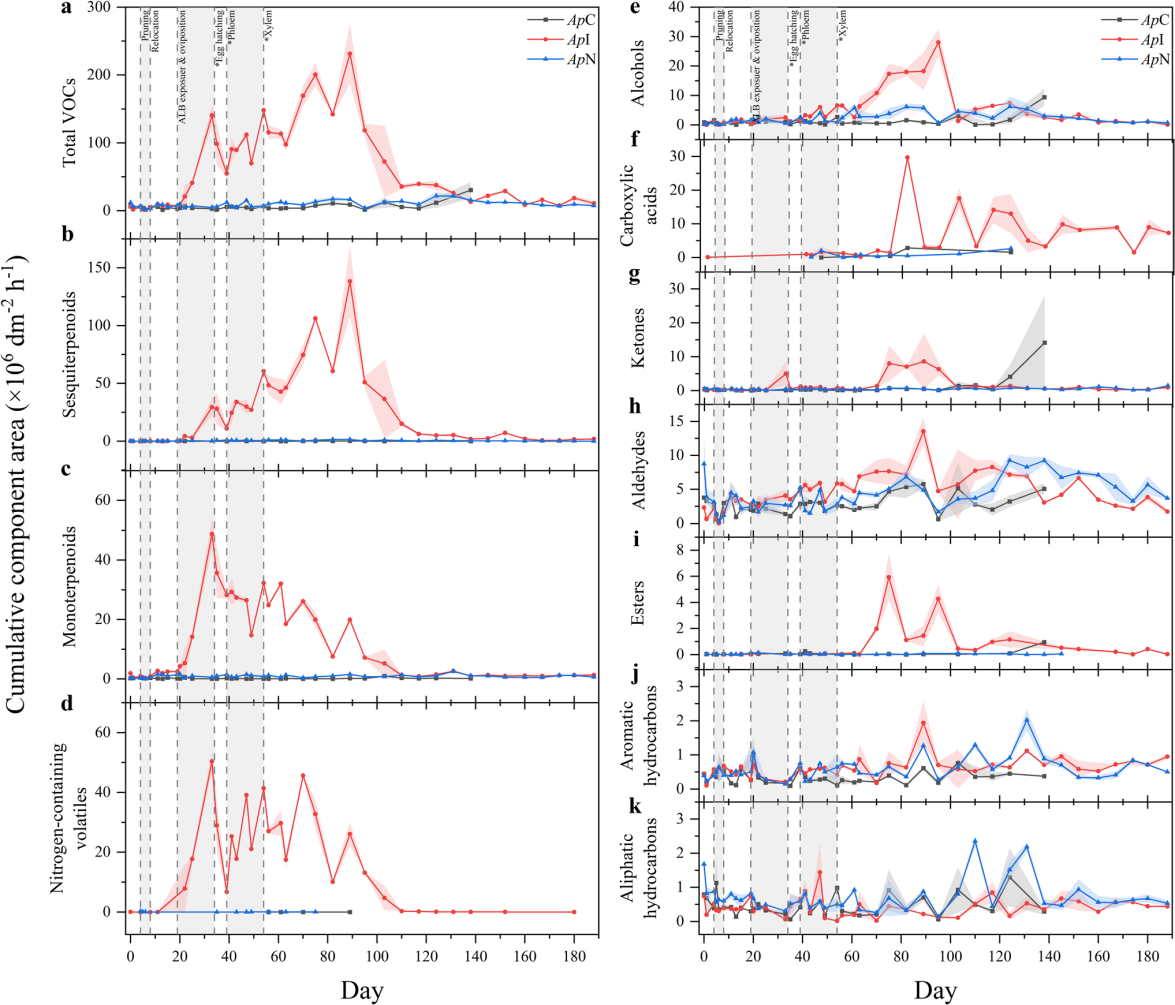
VOC emission from Acer platanoides trunks: non-infested control (ApC, black square), ALB-infested (ApI, red circle), and non-infested neighbor (ApN, blue triangle). Data shown are means ± standard errors of the means (shaded areas) of double determinations of the cumulative component areas of all 120 constitutive and induced plant volatiles (**a**), sesquiterpenoids (**b**), monoterpenoids (**c**), nitrogen-containing volatiles (**d**), alcohols (**e**), carboxylic acids (**f**), ketones (**g**), aldehydes (**h**), esters (**i**), aromatic hydrocarbons (**j**), and aliphatic hydrocarbons (**k**). Vertical dotted lines represent treatment steps and developmental stages of the ALB, estimated based on the temperature inside the quarantine room and literature data (Keena 2006; Keena and Moore 2010)

Total VOC emission rates differed considerably between treatments (Fig. 2). Following ALB infestation, emissions from *Ap*I increased substantially, showing an initial peak approximately two weeks after exposure and a pronounced maximum during the period coinciding with the estimated period of larvae feeding on xylem tissue (Fig. 2 a).

Terpenoids dominated emissions from *Ap*I in both diversity and emission strength (Fig. 2 b − c). Monoterpenoids (MTs; C_10_ − C_11_ terpenes and derivatives) and sesquiterpenoids (SQTs; C_15_ terpenes and derivatives) exhibited contrasting temporal patterns. Cumulative emissions of 23 MTs increased rapidly after infestation, peaked approximately two weeks post-exposure, and subsequently declined. In contrast, cumulative emissions of 26 SQTs increased more gradually, reaching a maximum around ten weeks after infestation. Thus, MTs dominated early responses associated with oviposition, whereas SQTs predominated during larval development.

Nitrogen-containing volatiles (NCVs; 11 compounds) represented the third largest contribution to total emissions from *Ap*I (Fig. 2 d) and were emitted almost exclusively from the infested maple. Additional compound classes that increased more strongly in *Ap*I than in *Ap*N or *Ap*C included alcohols (9 compounds), carboxylic acids (3), ketones (7), and esters (2). Aldehydes (11) increased only modestly in *Ap*I, while aliphatic and aromatic hydrocarbons were emitted at similar rates across all maples and contributed least to total VOC emissions despite their numerical diversity (17 aliphatic and 9 aromatic hydrocarbons), (Fig. 2 j − k).

### Constitutive Trunk Emissions

Trunk volatiles constitutively detected at all maple trees are summarized in Table 2 and were evaluated across three experimental phases: acclimation, abiotic stress (pruning and relocation), and biotic stress (ALB infestation). According to the Kruskal–Wallis rank sum test followed by Dunn’s post hoc test (*P* < 0.05), emission rates did not differ significantly among identically treated trees, allowing pooled analyses of all trees during acclimation phase and of *Ap*I and *Ap*N during abiotic stress phase (Table 1).

**Table 2:** Constitutive trunk emissions detected at the studied Norway maples (*Acer platanoides*, n = 3) and their change in emission rate upon abiotic and biotic stress. Only compounds detectable at all maples in ≥ 50% of the measurements (N = 60) within the first 20 days after arrival are included. Significance of emission changes among different health conditions was tested by Kruskal–Wallis rank sum test followed by Dunn’s post hoc test (*P* < 0.05). References comprise VOCs emitted from different parts of *Acer* (spp.) and with relevance to *Anoplophora glabripennis* Motschulsky (ALB), *D. helophoroides* (*D. h.*), and *Sclerodermus* (spp.) as attractant (A), repellent (R), neutral (N) or – depending on their concentration – both (A/R). Data on *Sclerodermus* (spp.) were supplemented by volatiles which showed binding affinities (BA) to odorant-binding proteins in antennae

LRI exp	MF	Compound ^a^	CAS	LRI lit	DF (%) (N = 60)	Contr. (%) ^b^	Significant difference ^c^				References					
											*Acer* (spp.)			Semiochemicals		
						M (SE)	S/H	I/H	N/H	I/N	Trunk	Canopy	Syrup	ALB	*D. h*	*Sclerodermus* (spp.)
		*Aldehydes*				*54.11 (1.44)*										
558	C_4_H_8_O	2-Methylpropanal	78—84—2	552	75	0.46 (0.03)		○●				(Zhang et al. 2008)	(Sabik et al. 2010)			
664	C_5_H_10_O	2-Methylbutanal^d^	96—17—3	662	82	0.90 (0.06)		○●	●	●			(Sabik et al. 2010)			
703	C_5_H_10_O	Pentanal^d^	110—62—3	700	67	1.79 (0.11)			○●			(Li et al. 2003; Zhang et al. 2008)				
804	C_6_H_12_O	Hexanal^d^	66—25—1	801	95	14.21 (0.66)	○	○	○●		(Makarow et al. 2020)	(Li et al. 2003; Hu et al. 2009)	(Sabik et al. 2010)			
839	C_7_H_14_O	2,2-Dimethylpentanal^M, R^	14,250—88—5	826	73	0.45 (0.01)										
906	C_7_H_14_O	Heptanal	111—71—7	901	82	2.73 (0.14)			●			(Zhang et al. 2008; Hu et al. 2009)	(Sabik et al. 2010)	A (Xu and Teale 2021)		BA (Huang et al. 2023)
967	C_7_H_6_O	Benzaldehyde^d^	100—52—7	962	75	4.38 (0.20)		○●	○●			(Zhang et al. 2008)	(Sabik et al. 2010)			BA (Huang et al. 2023)
1007	C_8_H_16_O	Octanal	124—13—0	1003	95	5.84 (0.45)						(Hu et al. 2009)	(Sabik et al. 2010)		N (Ren et al. 2017)	BA (Huang et al. 2023)
1107	C_9_H_18_O	Nonanal	124—19—6	1104	100	18.75 (1.10)		○			(Makarow et al. 2020)	(Li et al. 2003; Zhang et al. 2008; Hu et al. 2009; Wang et al. 2016)		A (Lyu et al. 2023)	A (Ren et al. 2017)	BA (Huang et al. 2023)
1209	C_10_H_20_O	Decanal^d^	112—31—2	1206	80	4.70 (0.39)		○		●		(Li et al. 2003; Zhang et al. 2008; Wang et al. 2016)				BA/N (Yi et al. 2018; Huang et al. 2023)
		*Aliphatic hydrocarbons*				*12.61 (0.18)*										
< 500	C_5_H_12_	2-Methylbutane^M, S^	78—78—4	475	90	0.73 (0.04)	○		●							
501	C_5_H_12_	Pentane	109—66—0	500	73	0.76 (0.02)										
602	C_6_H_14_	Hexane	110—54—3	600	72	1.13 (0.04)						(Zhang et al. 2008)				
701	C_7_H_16_	Heptane	142—82—5	700	100	1.32 (0.04)					(Makarow et al. 2020)	(Zhang et al. 2008)				
720	C_7_H_14_	Methylcyclohexane	108—87—2	727	60	0.29 (0.01)										
792	C_8_H_16_	1-Octene	111—66—0	789	85	0.74 (0.04)										
801	C_8_H_18_	Octane	111—65—9	800	100	3.51 (0.13)					(Makarow et al. 2020)	(Zhang et al. 2008)				
901	C_9_H_20_	Nonane	111—84—2	900	97	1.52 (0.07)		○	○							
1100	C_11_H_24_	Undecane	1120—21—4	1100	92	1.05 (0.04)			○		(Makarow et al. 2020)					BA (Huang et al. 2023)
1200	C_12_H_26_	Dodecane	112—40—3	1200	93	0.86 (0.04)					(Makarow et al. 2020)					
1400	C_14_H_30_	Tetradecane^d^	629—59—4	1400	70	0.74 (0.03)		○●	○		(Makarow et al. 2020)					BA (Huang et al. 2023)
		*Aromatic hydrocarbons*				*10.07 (0.17)*										
658	C_6_H_6_	Benzene	71—43—2	654	82	1.23 (0.02)					(Makarow et al. 2020)	(Ren et al. 2018)				
766	C_7_H_8_	Toluene	108—88—3	763	100	4.81 (0.12)		●	●		(Makarow et al. 2020)		(Sabik et al. 2010)			
871	C_8_H_10_	p-/m-Xylene^d^	106—42—3/ 108—38—3	865/ 866	100	2.00 (0.11)		●			(Makarow et al. 2020)		(Sabik et al. 2010)			N (Huang et al. 2023)
895	C_8_H_10_	o-Xylene	95—47—6	888	95	1.05 (0.04)		●	●							
997	C_9_H_12_	1,2,4-Trimethylbenzene ^d^	95—63—6	990	98	0.76 (0.02)	○●	○●	○●		(Makarow et al. 2020)					
1696	C_16_H_20_	Diisopropynaphthalene^M^	n.a. ^e^	1662–1728	63	0.24 (0.01)		○●	○●		(Makarow et al. 2020)					
		*Alcohols*				*9.30 (0.58)*										
519	C_3_H_8_O	Isopropyl alcohol	67—63—0	489	75	7.63 (0.57)					(Makarow et al. 2020)					
1035	C_8_H_18_O	2-Ethyl-1-hexanol^d^	104—76—7	1030	63	1.69 (0.07)		○●	○●			(Li et al. 2003; Zhang et al. 2008; Wang et al. 2016)	(Sabik et al. 2010)			
		*Ketones*				*7.28 (0.16)*										
509	C_3_H_6_O	Acetone	67—64—1	487	73	5.42 (0.14)					(Makarow et al. 2020)					
609	C_4_H_8_O	2-Butanone^d^	67—64—1	598	87	1.88 (0.09)		○●		●		(Li et al. 2003)	(Sabik et al. 2010)			
		*Monoterpenes*				*5.24 (0.18)*										
935	C_10_H_16_	α-Pinene^d^	80—56—8	937	100	2.41 (0.14)	○●	○●	○●	●	(Makarow et al. 2020)	(Li et al. 2003; Zhang et al. 2008; Wang et al. 2016; Ren et al. 2018)		A (Lyu et al. 2023)	A (Ren et al. 2017)	BA (Huang et al. 2023)
979	C_10_H_16_	β-Pinene^d^	127—91—3	979	78	0.59 (0.02)	○	○●	○●	●	(Makarow et al. 2020)	(Zhang et al. 2008; Wang et al. 2016)		A/R (Lyu et al. 2023)		BA (Huang et al. 2023)
1013	C_10_H_16_	3-Carene^d^	13,466—78—9	1011	93	1.71 (0.11)	○●	○●	○●		(Makarow et al. 2020)	(Zhang et al. 2008; Wang et al. 2016; Ren et al. 2018)		A (Lyu et al. 2023)	A (Ren et al. 2017)	BA (Huang et al. 2023)
1032	C_10_H_16_	Limonene^d^	138—86—3	1030	95	0.54 (0.01)	○	○●	○	●	(Makarow et al. 2020)	(Li et al. 2003; Zhang et al. 2008)		A (Lyu et al. 2023)	A (Ren et al. 2017)	BA/N (Yi et al. 2018; Huang et al. 2023)

LRI = linear retention index, MF = molecular formula, DF = detection frequency prior to ALB exposure.

^a^Identification methods: M = comparison of the mass spectrum with those contained in NIST20 Library, R = comparison of experimental LRI with literature LRI contained in NIST20 Library, S = Confirmation with analytical standards. If not otherwise stated (as a superscript letter), compounds were identified using all three methods (M, R, S).

^b^Contribution (%) was calculated as the proportion of the compound’s emission rate relative to the summed emission rate of all compounds listed in the table, with M = mean and SE = standard error of mean.

^c^Pair-wise comparison of health conditions: H = healthy, S = stressed, I = infested, N = neighboring to infestation.

● = Difference is significant for inter-tree comparisons.

○ = Difference is significant for intra-tree comparisons; not applicable for I vs. N.

**Fig. 3:**
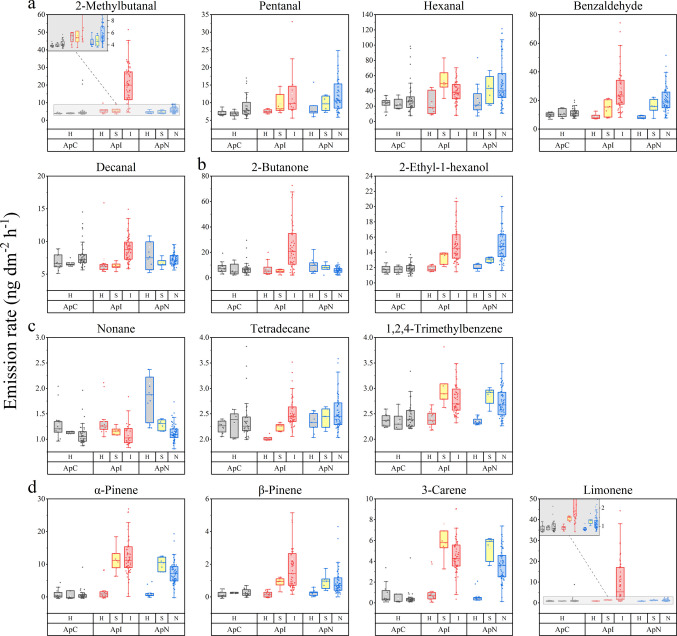
Box plot representation of quantitative changes in emission rates (ng dm^−2^ h^−1^) of constitutive volatiles emitted from *A. platanoides* trunks depending on health status with H = healthy (grey box), S = stressed (yellow box), I = ALB-infested (red box), and N = neighbored to infestation (blue box). The control tree (*Ap*C, black border) remained undamaged; *Ap*N (blue border) and *Ap*I (red border) were pruned and transported to the quarantine room, followed by infestation of *Ap*I with the ALB, and *Ap*N being noninfested but neighboring to *Ap*I. a. Aldehydes, b. Ketones and alcohols, c. Hydrocarbons, and d. Monoterpenoids

^d^Box plots are visualized in Fig. 3.

^e^Isomers were not separable or unknown.

Aldehydes dominated the constitutive volatile blend, particularly saturated C_5_–C_10_ aldehydes and benzaldehyde, with hexanal and nonanal as major components (Table 2). Emissions of most aldehydes declined during acclimation but increased following relocation of *Ap*I and *Ap*N, becoming significant immediately after relocation for hexanal and under prolonged stress conditions for most other aldehydes. The only aldehydes that exhibited significant changes in emission rates upon infestation-induced biotic stress were 2-methylbutanal and decanal. Both rates were elevated by a factor of 3.5 and 1.2, respectively, as displayed in Fig. 3 a, comparing *Ap*I and *Ap*N in the third experimental phase.

Aliphatic and aromatic hydrocarbons represented the second most abundant class of constitutive trunk volatiles and consisted mainly of C_5_–C_14_ alkanes and alkyl benzenes. Emissions of most aliphatic hydrocarbons declined over time, as exemplified for nonane (Fig. 3 c), whereas tetradecane increased gradually. Some aromatic hydrocarbons such as 1,2,4-trimethylbenzene increased under stress conditions (Fig. 3 c), but neither aliphatic nor aromatic hydrocarbons responded significantly to ALB infestation.

Two alcohols and two ketones were consistently detected at all maple trees. Among these, 2-butanone increased significantly following ALB-infestation, with emission rates from *Ap*I approximately fourfold higher than from noninfested trees (Fig. 3 b), whereas 2-ethyl-1-hexanol responded to abiotic stress but remained unaffected by infestation.

Among all compound classes contributing to constitutive trunk emissions, MTs were the most sensitive to stress treatments. Emissions of all MTs listed in Table 2 increased significantly in *Ap*I and *Ap*N following relocation to the quarantine facility. The strongest increases were observed for α-pinene and 3-carene (6 − ninefold), followed by β-pinene (4–sixfold) and limonene (~ 1.5-fold) (Fig. 3 d). Responses to direct and neighboring ALB herbivory varied among individual MTs. Emissions of 3-carene declined slightly in both *Ap*I and *Ap*N, whereas other MTs decreased in *Ap*N but remained stable or increased in *Ap*I. Limonene showed the strongest response to infestation, with mean emission rates approximately eightfold higher in *Ap*I than in *Ap*N.

### Herbivore-Induced Plant Volatiles

Volatiles that appeared de novo or increased in *Ap*I following ALB infestation and differed in emission rates relative to non-infested maples were classified as herbivore-induced plant volatiles (HIPVs). 36 compounds were detected in ≥ 50% of *Ap*I’s measurements after ALB exposure (Table 3), and an additional 32 compounds in ≥ 30% (Table S1). HIPVs consisted predominantly of terpenoids (65%), NCVs (16%) and oxygen-containing volatiles (OCVs), including alcohols, carboxylic acids, esters, and ketones, each contributing less than 5%.

**Table 3:** Herbivore-induced plant volatiles emitted from the trunk of a Norway maple (*Acer platanoides*) infested with the Asian longhorned beetle (ALB, *Anoplophora glabripennis* Motschulsky), detected in ≥ 50% of the measurements within the first 24 weeks after ALB exposure. References comprise VOCs emitted from different parts of *Acer* (spp.) and with relevance to ALB, *D. helophoroides* (*D. h.*), and *Sclerodermus* (spp.) as attractant (A), repellent (R), neutral (N) or – depending on their concentration – both (A/R). Data on ALB were supplemented by data of electroantennographic (EAG) responses and those on *Sclerodermus* (spp.) by data of binding affinities (BA) to odorant-binding proteins in antennae

LRI exp	MF	Compound name ^a^	CAS	NIST20		DF (%) (N = 61)	Emission rate ratio ^b^			Contr (%) ^c^	References					
				SM	LRI lit		Inter-tree		Intra-tree		*Acer* (spp.)			Semiochemicals		
							*Ap*I*/* *Ap*N	*Ap*I*/* *Ap*C	pre/post ALB	M (SE)	Trunk	Canopy	Syrup	ALB	*D. h*	*Sclerodermus* (spp.)
		*Mono- and homoterpenoids*								*22.1 (1.2)*						
924	C_10_H_16_	Tricyclene ^M, R^	508—32—7	91 (3)	925	57	>	>	ALB	0.22 (0.03)						
930	C_10_H_16_	α-Thujene ^M, R^	2867—05—2	89 (5)	929	56	*Ap*I	*Ap*I	>	0.07 (0.01)		(Zhang et al. 2008)				
950	C_10_H_16_	Camphene	79—92—5	91 (8)	952	74	41	>	57	2.24 (0.39)	(Makarow et al. 2020)	(Zhang et al. 2008; Ren et al. 2018)		A (Lyu et al. 2023)		BA (Huang et al. 2023)
976	C_10_H_16_	Sabinene	3387—41—5	92 (6)	974	64	>	>	19	1.32 (0.12)		(Zhang et al. 2008)				
979	C_10_H_16_	β-Pinene	127—91—3	85 (6)	979	98	2	6	4	0.28 (0.04)	(Makarow et al. 2020)	(Zhang et al. 2008; Wang et al. 2016)		A/R (Lyu et al. 2023)		
994	C_10_H_16_	β-Myrcene	123—35—3	90 (7)	991	61	>	*Ap*I	17	0.91 (0.09)		(Ren et al. 2018)		R (Lyu et al. 2023)		BA (Huang et al. 2023)
1028	C_10_H_14_	p-Cymene	99—87—6	87 (8)	1025	98	117	93	34	5.73 (0.84)		(Ren et al. 2018)			A (Yi et al. 2023)	
1032	C_10_H_16_	Limonene	138—86—3	88 (6)	1030	98	14	14	16	1.02 (0.15)	(Makarow et al. 2020)	(Li et al. 2003; Zhang et al. 2008)		A_f_ (Lyu et al. 2023)	A (Wei et al. 2008; Ren et al. 2017)	BA/N (Yi et al. 2018; Huang et al. 2023)
1052	C_10_H_16_	β-Ocimene	3338—55—4	94 (5)	1038	57	>	>	4	1.40 (0.21)		(Zhang et al. 2008; Wang et al. 2016; Ren et al. 2018)		R (Lyu et al. 2023)		
1062	C_10_H_16_	γ-Terpinene	99—85—4	96 (5)	1060	61	*Ap*I	*Ap*I	89	3.17 (0.29)		(Zhang et al. 2008; Wang et al. 2016)			A (Yi et al. 2023)	
1119	C_11_H_18_	(*E*)−4,8-Dimethylnona-1,3,7-triene ^M, R^	51,911—82—1	88 (7)	1114	66	73	*Ap*I	18	5.21 (0.63)		(Ren et al. 2018)				
1240	C_11_H_16_O	Thymol methyl ether ^M, R^	1076—56—8	93 (2)	1235	57	*Ap*I	*Ap*I	ALB	0.35 (0.04)						
1250	C_11_H_16_O	Carvacrol methyl ether ^M, R^	6379—73—3	90 (5)	1244	51	*Ap*I	*Ap*I	ALB	0.16 (0.02)						
		*Sesquiterpenoids*								*40.2 (2.7)*						
1361	C_15_H_24_	α-Longipinene	5989—08—2	93 (6)	1353	95	93	141	ALB	8.36 (1.36)	(Makarow et al. 2020)			A (Xu et al. 2020)		A (Yi et al. 2018)
1378	C_15_H_24_	Cyclosativene ^M, R^	22,469—52—9	95 (3)	1368	95	>	146	ALB	8.93 (1.33)	(Makarow et al. 2020)					
1385	C_15_H_24_	α-Copaene	3856—25—5	95 (2)	1376	95	>	>	ALB	5.08 (0.90)	(Makarow et al. 2020)			EAG (Xu et al. 2020)		
1431	C_15_H_24_	(*E*)-β-Caryophyllene	87—44—5	93 (4)	1419	72	*Ap*I	*Ap*I	6	0.65 (0.10)	(Makarow et al. 2020)	(Zhang et al. 2008; Wang et al. 2016; Ren et al. 2018)		A/R (Lyu et al. 2023)	R (Ren et al. 2017)	BA (Huang et al. 2023)
1444	C_15_H_24_	(*E*)-α-Bergamotene	13,474—59—4	92 (3)	1435	72	3	>	ALB	0.28 (0.04)				EAG (Xu et al. 2020)		
1462	C_15_H_24_	epi-β-Caryophyllene ^M, R^	68,832—35—9	93 (2)	1466	57	>	*Ap*I	ALB	0.45 (0.07)						
1474	C_15_H_24_	Alloaromadendrene ^M, R^	25,246—27—9	92 (3)	1461	57	*Ap*I	*Ap*I	ALB	0.26 (0.05)		(Ren et al. 2018)				
1491	C_15_H_22_	α-Curcumene	644—30—4	91 (7)	1483	95	93	>	ALB	7.88 (1.14)	(Makarow et al. 2020)					
1503	C_15_H_24_	Zingiberene	495—60—3	90 (8)	1495	84	*Ap*I	>	ALB	6.00 (1.17)	(Makarow et al. 2020)					
1517	C_15_H_24_	Unknown sesquiterpene	n.a	n.a	n.a	79	*Ap*I	*Ap*I	ALB	1.09 (0.17)						
1534	C_15_H_24_	β-Sesquiphellandrene	20,307—83—9	90 (8)	1524	90	*Ap*I	*Ap*I	ALB	1.27 (0.22)						
		*Nitrogen-containing compounds*								*29.4 (1.8)*						
628	C_4_H_7_N	Isobutyronitrile	78—82—0	96 (3)	626	61	*Ap*I	*Ap*I	ALB	0.76 (0.12)						
681	C_5_H_11_NO	Butanal *O*-methyloxime ^M, R^	31,376—98—4	87 (2)	680	57	*Ap*I	*Ap*I	ALB	5.46 (0.70)						
688	C_5_H_11_NO	Butanone *O*-methyloxime ^M, R^	n.a	83 (3)	687	59	>	*Ap*I	ALB	15.24 (1.52)						
703	C_5_H_7_N	2-Methyl-3-butenenitrile ^M^	16,529—56—9	92 (4)	n.a	56	*Ap*I	*Ap*I	ALB	1.02 (0.11)						
728	C_5_H_9_N	2-Methylbutanenitrile ^M, R^	18,936—17—9	90 (8)	723	74	>	>	>	3.65 (0.42)						
861	C_6_H_11_NO	Unknown oxime 2	n.a	81 (1)	n.a	57	*Ap*I	*Ap*I	ALB	1.37 (0.14)						
865	C_6_H_11_NO	Unknown oxime 3	n.a	78 (1)	n.a	54	*Ap*I	*Ap*I	ALB	1.95 (0.21)						
		*Oxygen-containing compounds*								*8.2 (0.9)*						
503	C_2_H_6_O	Ethanol ^M, R^	64—17—5	89 (6)	427	59	14	8	59	2.77 (0.86)						
607	C_4_H_8_O	2-Butanone	78—93—3	88 (7)	598	85	6	5	6	0.43 (0.06)		(Li et al. 2003)	(Sabik et al. 2010)			
664	C_5_H_10_O	2-Methylbutanal	96—17—3	93 (4)	662	93	7	9	8	0.72 (0.06)			(Sabik et al. 2010)			
704	C_2_H_4_O_2_	Acetic acid	64—19—7	96 (4)	610	57	3	>	ALB	2.34 (0.29)			(Sabik et al. 2010)			
746	C_5_H_12_O	2-Methyl-1-butanol ^M, R^	137—32—6	94 (5)	739	89	>	7	>	1.99 (0.23)						

LRI = linear retention index, MF = molecular formula, SM = spectral match, given as mean (standard deviation), DF = detection frequency after ALB exposure, *Ap*I = ALB-infested, *Ap*N = neighboring to ALB-infestation, *Ap*C = control.

^a^Identification methods: M = comparison of the mass spectrum with those contained in NIST20 Library, R = comparison of experimental LRI with literature LRI contained in NIST20 Library, S = Confirmation with analytical standards. If not otherwise stated (as a superscript letter), compounds were identified using all three methods (M, R, S).

^b^For inter-tree comparison, ratio was calculated from component areas averaged over period 3 (after *Ap*I has been exposed to ALB), and for *Ap*I intra-tree comparison, ratio was calculated from component areas averaged over period 1–2 (N = 20) and 3 (N = 61), respectively. Only component areas of compounds that were detected in at least 20% of measurements per sample type and period were used for calculation. Inter-tree ratios of compounds are marked with ‘*Ap*I ‘ if they were exclusively detected at *Ap*I, and with ‘ > ’ if they appeared in less than 20% of measurements of the noninfested maples. Intra-tree ratios are marked with ‘ALB’ if the respective compound appeared de novo after ALB exposure or in less than 20% of measurements prior ALB exposure.

^c^Contribution (%) was calculated as the proportion of the compound’s emission rate relative to the summed emission rate of all compounds listed in the table, with M = mean and SE = standard error of mean.

With 13 individual compounds, MTs constituted the most numerous class of HIPVs. Compounds already present prior to infestation but strongly upregulated upon herbivory contributed most to total MT emissions, whereas de novo MTs accounted for less than 4%. The largest contributors to total MT emissions were *p*-cymene (26%), the homoterpene (*E*)−4,8-dimethylnona-1,3,7-triene (DMNT; 24%), γ-terpinene (14%), and camphene (10%).

**Fig. 4:**
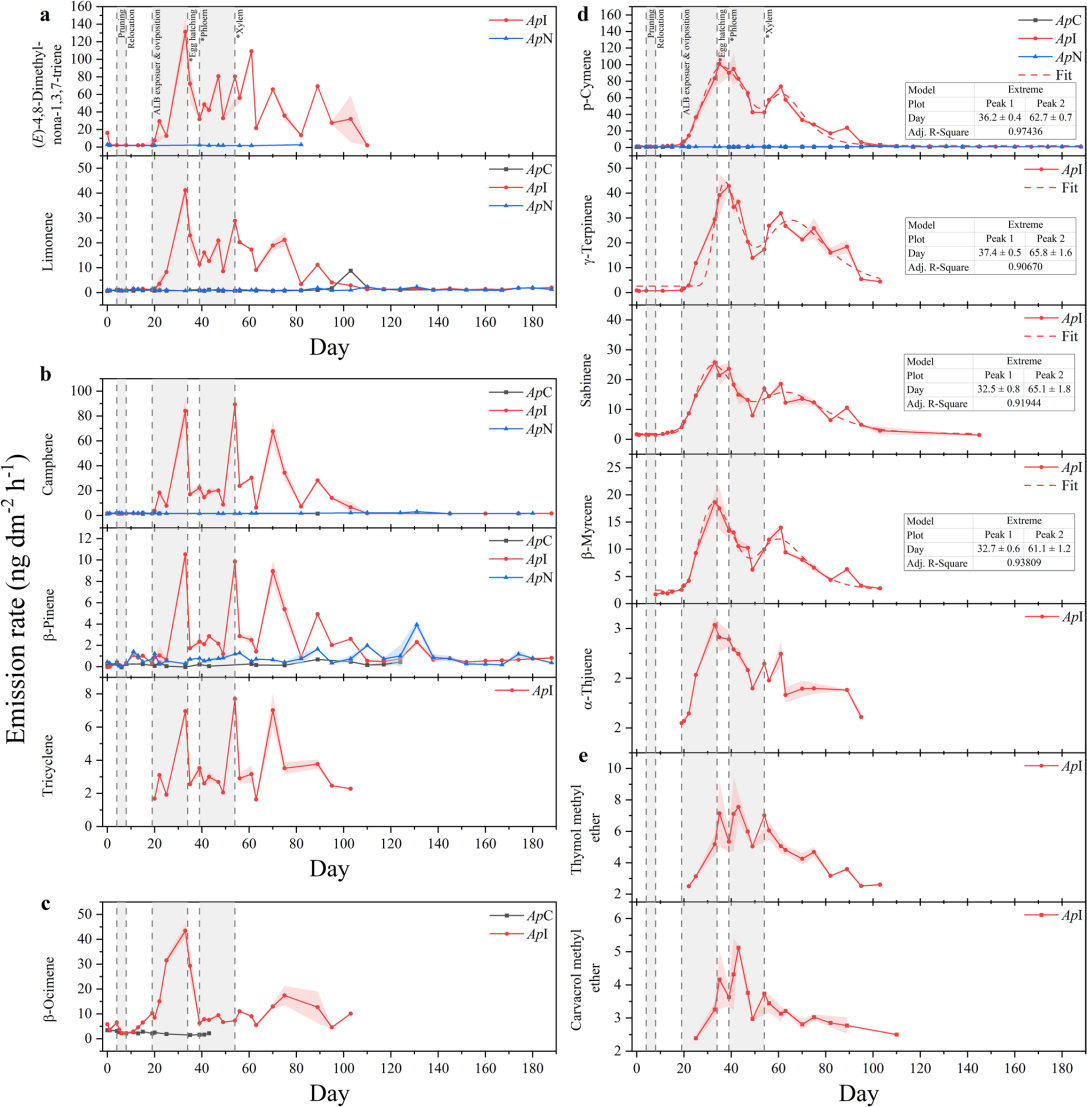
Temporal emission dynamics of herbivore-induced C_10_–C_11_ terpenoids, emitted from trunks of an ALB-infested *A. platanoides* (*Ap*I, red circle), its noninfested neighbor (*Ap*N, blue triangle), and a non-infested control (*Ap*C, black square). Data shown are means ± standard error or means (shaded areas) of double determinations and compounds were grouped according to their temporal emission patterns and sorted by emission rate. Vertical dotted lines represent treatment steps and developmental stages of the ALB, estimated based on the temperature inside the quarantine room and literature data (Keena 2006; Keena and Moore 2010). Data of p-cymene (Adj. R^2^ = 0.97435), γ-terpinene (Adj. R^2^ = 0.90670), sabinene (Adj. R^2^ = 0.91944), and β-myrcene (Adj. R^2^ = 0.93809) were fitted with the peak fitting function ‘Extreme’ by OriginLab

Based on Kendall’s Tau correlation coefficients τ (Fig. S3), MT emission dynamics were grouped into four characteristic temporal patterns. DMNT and limonene (τ = 0.64, *P* < 0.001) closely followed the cumulative MT emission pattern, peaking shortly before larval hatching and subsequently declining (Fig. 4 a). Camphene, β-pinene, and tricyclene (τ = 0.66–0.84, *P* < 0.001) increased during oviposition but, unlike DMNT and limonene, exhibited three distinct emission peaks (Fig. 4 b), coinciding with the estimated onsets of egg hatching, xylem feeding, and approximately two weeks thereafter. β-Ocimene did not correlate with any other monoterpenoid (τ < 0.30, not significant), instead it showed the earliest infestation-induced increase, remained elevated throughout oviposition, and declined when larvae likely began feeding on phloem and xylem tissues (Fig. 4 c). The most distinct dynamics were observed for p-cymene, γ-terpinene, sabinene, β-myrcene, and α-thujene (τ = 0.58–0.85, *P* < 0.001) (Fig. 4 d), which displayed two emission peaks coinciding with the estimated periods of egg hatching and early xylem feeding. Thymol methyl ether and carvacrol methyl ether did not conform clearly to any pattern but most closely correlated with the MTs showing two-peak dynamics (τ = 0.43–0.67, *P* < 0.05) (Fig. 4 e).

In contrast to MTs, nearly all SQTs appeared de novo following ALB infestation. SQT emissions were dominated by cyclosativene, α-longipinene, α-curcumene, zingiberene, and α-copaene, together accounting for approximately 90% of total SQT emissions. Except for zingiberene, all SQTs were also detected at noninfested maples, albeit at substantially lower detection frequencies and emission rates.

**Fig. 5:**
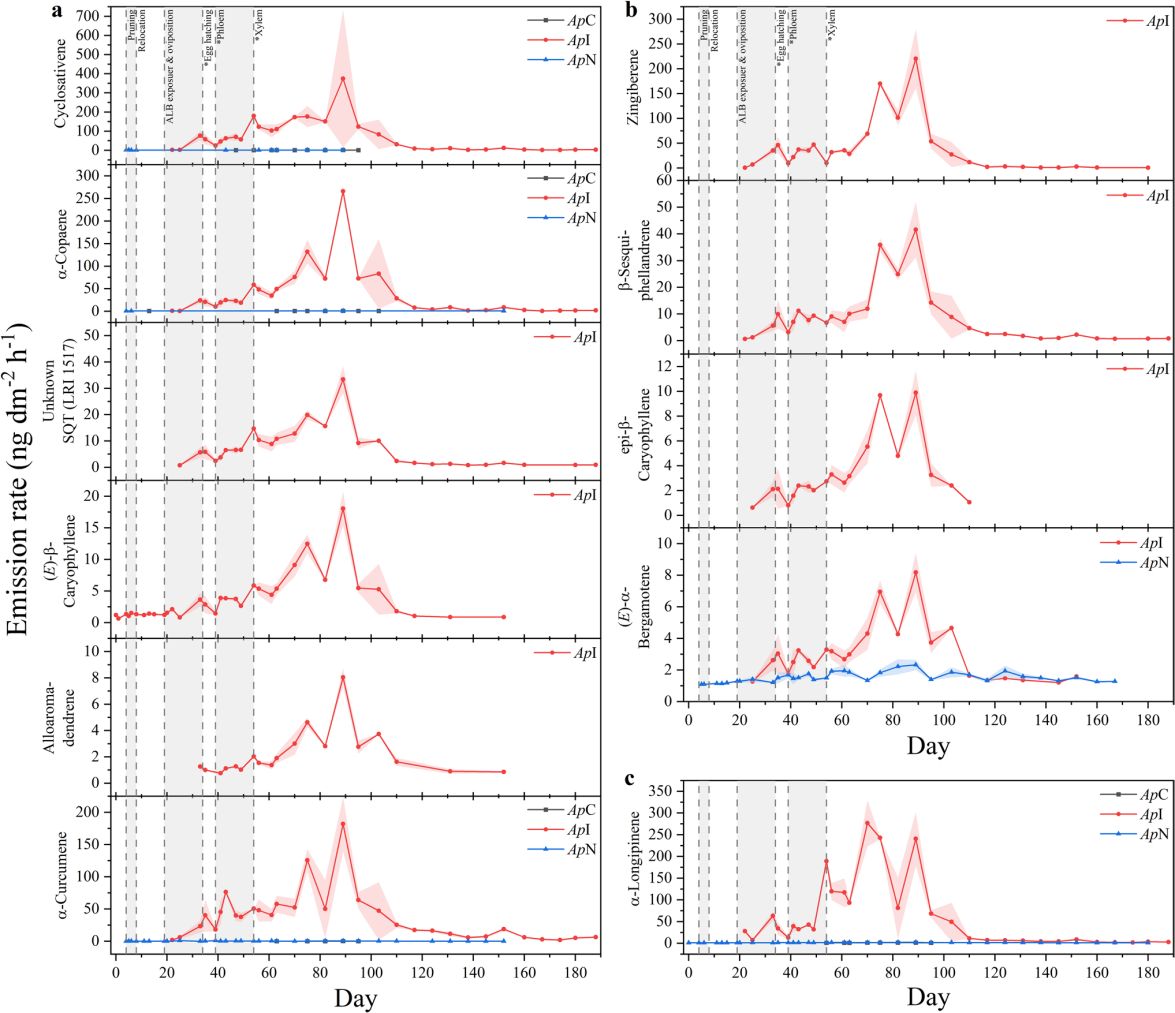
Temporal emission dynamics of herbivore-induced sesquiterpenoids, emitted from trunks of an ALB-infested *A. platanoides* (*Ap*I, red circle), its noninfested neighbor (*Ap*N, blue triangle), and a non-infested control (*Ap*C, black square). Data shown are means ± standard errors of means (shaded areas) of double determinations. Vertical dotted lines represent treatment steps and developmental stages of the ALB, estimated based on the temperature inside the quarantine room and literature data (Keena 2006; Keena and Moore 2010)

SQT emission dynamics were less variable than those of MTs and, expect for the pairwise comparison of zingiberene and alloaromadendrene (τ = 0.21, not significant), all SQTs showed moderate to strong correlation with each other (τ = 0.37–0.87, *P* < 0.05, Fig. S4). Although a clear classification into distinct groups based on Kendall’s τ was not possible, a tentative grouping into two overlapping patterns was proposed. The first group – including cyclosativene, α-copaene, (*E*)-β-caryophyllene, alloaromadendrene, α-curcumene, and an unknown SQT – closely followed the cumulative SQT emission pattern, peaking approximately ten weeks post-exposure (Fig. 5 a). Minor early-stage deviations included increases coinciding with the estimated period of xylem feeding (*e.g.*, cyclosativene) or transient peaks associated with the estimated period of phloem feeding (*e.g.*, α-curcumene). The second group – zingiberene, β-sesquiphellandrene, epi-β-caryophyllene, and (*E*)-α-bergamotene – exhibited relatively constant emissions during the estimated periods of oviposition and early-stage larvae, followed by two pronounced peaks approximately three and six weeks after larvae likely entered xylem tissue (Fig. 5 b). Despite showing moderate to strong correlation with other SQTs, α-Longipinene did not conform to either pattern but dominated SQT emissions during the first few days after ALB exposure, showed the steepest increase at the estimated onset of xylem feeding, and exhibited three pronounced peaks (Fig. 5 c). Although α-longipinene, (*E*)-α-bergamotene, and α-curcumene were also frequently detected at noninfested maples, their emission strengths were substantially lower and temporal dynamics differed from those observed in *Ap*I.

Beyond terpenoids, NCVs contributed substantially to the long-term HIPV blend of *Ap*I (Fig. 2 d). Due to limited representation in NIST 20 and the absence of literature retention indices, most NCVs could only be tentatively identified or assigned to compound classes. Except for 2-methylbutanenitrile, NCVs were exclusive to *Ap*I and occurred primarily during the first 13–14 weeks after infestation.

**Fig. 6:**
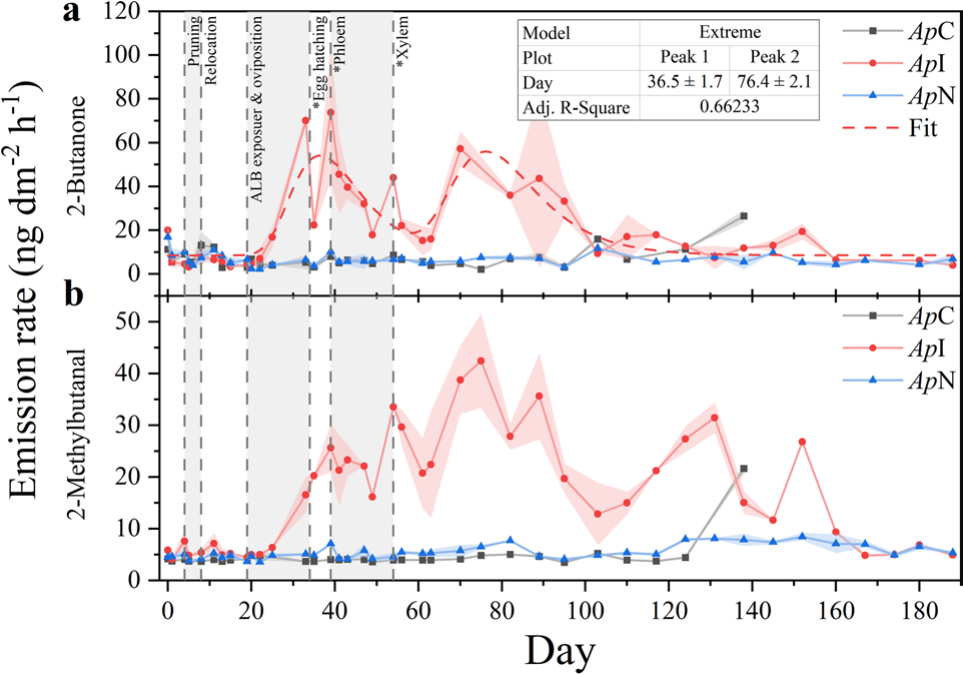
Temporal emission dynamics of herbivore-induced oxygen-containing volatiles, emitted from trunks of an ALB-infested A. platanoides (ApI, red circle), its noninfested neighbor (ApN, blue triangle), and a non-infested control (ApC, black square). Data shown are means ± standard errors of means (shaded areas) of double determinations. Vertical dotted lines represent treatment steps and developmental stages of the ALB, estimated based on the temperature inside the quarantine room and literature data (Keena 2006; Keena and Moore 2010). Data of 2-butanone (Adj. R^2^ = 0.66233) were fitted with the peak fitting function ‘Extreme’ by OriginLab

Last, several OCVs were classified as long-term HIPVs, which were detected also at noninfested maples. Ethanol, 2-butanone, and 2-methylbutanal occurred at similar frequencies across all maples, whereas acetic acid and 2-methyl-1-butanol were detected more frequently in *Ap*I. Distinct emission dynamics were only observed for 2-butanone, 2-methyl-1-butanol and 2-methylbutanal. 2-Butanone exhibited two emission peaks (Fig. 6 a), broadly comparable to those of some MTs, yet lacking strong correlations (τ < 0.5, *P* < 0.05). Emission dynamics of 2-methylbutanal and 2-methyl-1-butanol correlated moderately (τ = 0.49, *P* < 0.01), increased during the estimated periods of oviposition and early larval development, and peaked two to three weeks after the estimated onset of larvae feeding on xylem tissue (Fig. 6 b). Acetic acid was first detected when larvae likely began feeding on phloem tissue and increased steadily, whereas ethanol emission was high for only three weeks during estimated larval feeding in xylem tissue.

### Induced Changes in the Neighboring Tree

The noninfested maple (*Ap*N) emitted several volatiles that increased or appeared de novo following infestation of the adjacent maple (*Ap*I). Three sesquiterpenes – *(Z)*-α-bergamotene, α-santalene, and cuparene – and one ketone (2-heptanone) were detected in ≥ 50% of *Ap*N’s measurements and were emitted either exclusively by *Ap*N or at rates more than twice as high as those from both *Ap*I and *Ap*C (Table S2). In addition, the SQTs α-longipinene, (*E*)-α-bergamotene, and α-curcumene, previously identified as HIPVs, also showed elevated emissions in *Ap*N, although increases were less pronounced than in *Ap*I.

**Fig. 7:**
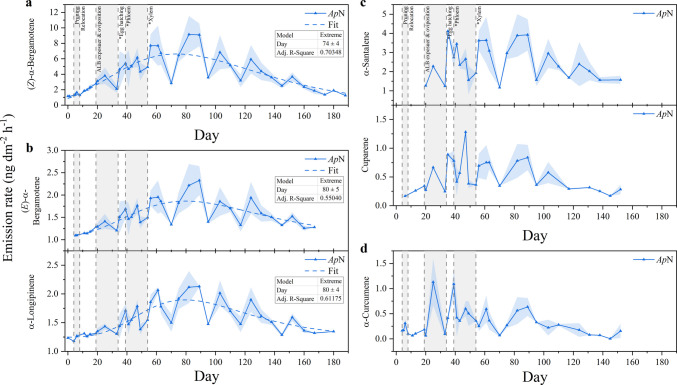
Temporal emission dynamics of sesquiterpenoids, emitted from trunks of a (noninfested) Norway maple (*A. platanoides, Ap*N), neighbored to an ALB-infested maple (*Ap*I). Data shown are means ± standard errors of means (shaded areas) of double determinations. Vertical dotted lines represent treatment steps to *Ap*I and developmental stages of the ALB, estimated based on the temperature inside the quarantine room and literature data (Keena 2006; Keena and Moore 2010). Data of (*Z*)-α-bergamotene (Adj. R^2^ = 0.70348), (*E*)-α-bergamotene (Adj. R^2^ = 0.55040), and α-longipinene (Adj. R^2^ = 0.61175) were fitted with the peak fitting function ‘Extreme’ by OriginLab

Temporal emission patterns differed among compounds. Whereas 2-heptanone increased steadily over time, SQT emissions exhibited distinct dynamics that could be grouped into two correlating clusters. Emissions of *(Z)*-α-bergamotene followed a peak-shaped pattern with a maximum being reached approximately eight weeks after infestation of the adjacent *Ap*I (Fig. 7 a) and correlated strongly with those of α-longipinene and *(E)*-α-bergamotene at *Ap*N (τ = 0.81 − 0.86, *P* < 0.001, Fig. 7 b). In contrast, α-santalene and cuparene – correlating with α-curcumene (τ = 0.61 − 0.65, *P* < 0.001) – deviated from these emission profiles during the early stages of infestation, showed elevated emissions shortly after infestation of *Ap*I (Fig. 7 c − d). However, their patterns converged once larvae likely began feeding on xylem tissue.

An additional twelve volatiles increased in emission rates from *Ap*N and were detected in ≥ 30% of measurements, including three ketones, five hydrocarbons, two alcohols, and one furan (Table S2). Owing to their low detection frequencies, their emission profiles were less well defined than those of the SQTs. Nevertheless, emissions of 1-penten-3—ol and 2,4-dimethyl-1-heptene increased transiently approximately 15–22 weeks and 12–17 weeks, respectively, after infestation of the adjacent *Ap*I.

### Stress-Induced Plant Volatiles

**Fig. 8:**
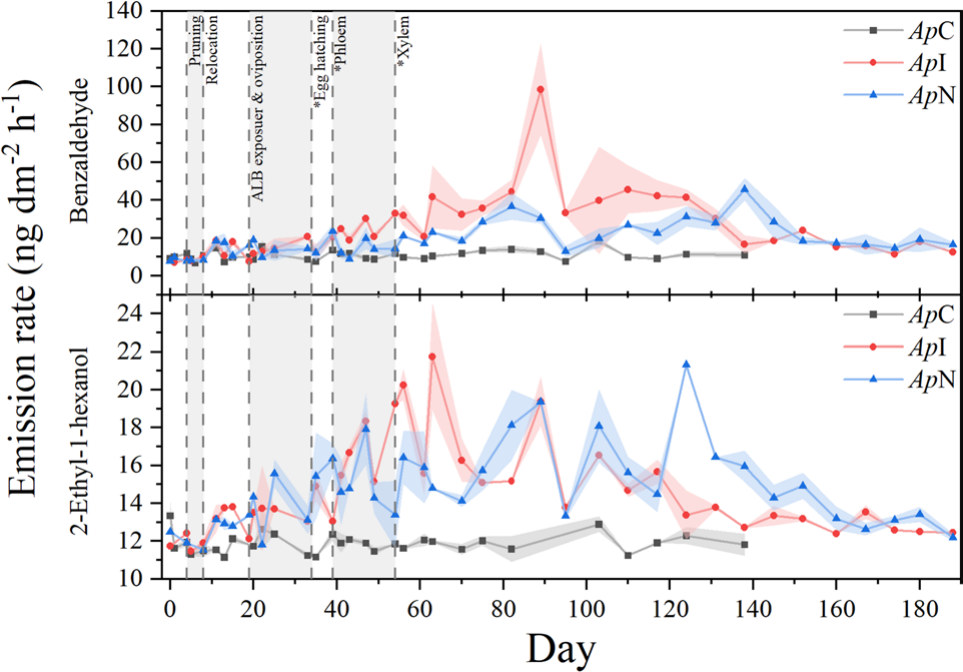
Temporal emission dynamics of trunk volatiles emitted from an ALB-infested *A. platanoides* (*Ap*I, red circle), its noninfested neighbor (*Ap*N, blue triangle), and a non-infested control (*Ap*C, black square). Data shown are means ± standards errors of means (shaded areas) of double determinations. Vertical dotted lines represent treatment steps and developmental stages of the ALB, estimated based on the temperature inside the quarantine room and literature data (Keena 2006; Keena and Moore 2010)

Last, six compounds were detected in ≥ 50% of measurements that increased similarly in both *Ap*I and *Ap*N compared to *Ap*C outside the quarantine facility (Table S3). Beside the constitutive trunk volatiles 2-ethyl-1-hexanol and benzaldehyde, stress-induced volatiles included benzyl alcohol, 3-methylbutanal, and the aromatic hydrocarbons styrene and propylbenzene. Emission rates of 2-ethyl-1-hexanol, benzyl alcohol, and propylbenzene were similar between *Ap*I and *Ap*N, whereas 3-methylbutanal, benzaldehyde, and styrene were emitted at slightly higher rates from *Ap*I. All of their temporal emission patterns showed a more or less pronounced peak-shape, with a maximum usually being reached earlier in *Ap*I than in *Ap*N (Fig. 8).

## Discussion

VOC emissions from infested plants comprise constitutive volatiles that are continuously produced and emitted and induced volatiles that are synthesized or upregulated in response to herbivory (Chen 2008). In the present study, constitutive and induced trunk volatiles emitted from an ALB-infested *A. platanoides* trunk showed only limited overlap. Four constitutive volatiles increased upon herbivory, whereas most HIPVs were either produced de novo or occurred at much lower abundances in noninfested maples. Furthermore, constitutive and induced volatile blends differed markedly in the compound classes contributing to total VOC emissions, indicating distinct regulatory mechanisms.

Despite their compositional differences, both constitutive and induced plant volatiles may function as semiochemicals for herbivores and their natural enemies. While many studies focus exclusively on HIPVs, several have demonstrated that blends of constitutive and induced volatiles can be more effective in mediating trophic interactions than HIPVs alone. For example, blends of constitutive and induced volatiles attracted the parasitic wasps *Cotesia marginiventris* and *Chelonus insularis*, parasitizing herbivorous lepidopteran larvae feeding on maize, more effectively than HIPVs alone (Fontana et al. 2011; Ortiz-Carreon et al. 2019). These findings underscore the importance of considering entire volatiles profiles, rather than isolated fractions, when evaluating chemical communication in multitrophic systems.

Constitutive trunk emissions from three *A. platanoides* trees were dominated in this study by aldehydes, with contributions from hydrocarbons, alcohols, ketones, and monoterpenoids (MT). Many of these compounds have previously been reported as emissions from various *Acer* tissues (Table 2). As data on *A. platanoides* are limited, comparisons were also made with studies on other *Acer* species. However, it is worth noting that VOC profiles can vary substantially among species, even within the same plant family. For example, although leaf emissions of *A. platanoides, A. negundo*, *A. mono*, and *A. truncatum* were all dominated by the green-leaf volatile (GLV) *(E)*−3-hexenyl acetate, their overall emission profiles differed markedly (Zhang et al. 2008). Even greater inter-species variability has been described for *Betula* (spp.), with *B. pendula* emissions being dominated by GLVs, and *B. pubescens* by MTs and SQTs (Zhang et al. 1999). Such variability may contribute to host susceptibility to ALB infestation, as reported for *Acer* (spp.) and *Populus* (spp.) (Wang et al. 2023). Furthermore, inconsistencies are also apparent among studies of the same species. For instance, Zhang et al*.* identified *(E)*−3-hexenyl acetate and acetic acid hexyl ester as dominant emissions from *A. negundo* leaves (Zhang et al. 2008), whereas Li et al*.* identified butyl acetate, 2-ethyl-1-hexanol, and nonanal as major compounds (Li et al. 2003). These discrepancies complicate direct comparisons and highlight the influence of methodological differences, plant condition, and environmental factors on VOC profiles.

The closest resemblance of constitutive trunk volatiles was observed with previously reported emissions from ALB-infested trunks (Makarow et al. 2020). Differences particularly within the aldehyde group may partly reflect analytical scopes, as Makarow et al*.* targeted ALB-associated compounds rather than entire VOC profiles. Nevertheless, most aldehydes detected here have been reported as emissions from *Acer* leaves (Li et al. 2003; Zhang et al. 2008; Hu et al. 2009; Wang et al. 2016) and maple syrup (Sabik et al. 2010), suggesting that these compounds may be transported through the xylem and diffuse outward through cambial tissues. Although several compounds were common to both trunk and leaf emissions, their relative abundances differed substantially. Leaf emissions of *A. platanoides* are dominated by GLVs, particularly *(E)*−3-hexenyl acetate and *(Z)*−3-hexen-1—ol (Zhang et al. 2008), neither of which were detected in trunk emissions. Conversely, hexanal and nonanal together accounted for more than 30% of constitutive trunk emissions but contributed less than 6% to leaf emissions (Zhang et al. 2008). Among all compound classes, MTs showed the highest consistency across tissues. α-Pinene, β-pinene, 3-carene, and limonene have been reported in nearly all *Acer* VOC studies (Li et al. 2003; Zhang et al. 2008; Hu et al. 2009; Wang et al. 2016), with comparable relative contributions to total VOC emission between trunks and leaves.

Considering the pronounced tissue specificity of VOC profiles, comparisons with trunk emissions from other deciduous trees would be beneficial. However, noninvasive studies quantifying constitutive trunk emissions from *Acer* or other ALB host species are currently lacking. Comparisons with studies using mechanically removed trunk tissues indicate partial similarity but also notable differences, potentially due to wound-related emissions. Among tree species susceptible to ALB infestation, *A. platanoides* showed the greatest resemblance to *Castanea sativa* (Fagaceae) and *Populus alba* var. *pyramidalis* Bunge (Salicaceae), both of which emit aldehyde-dominated blends (Özgenç et al. 2017; Gao et al. 2018). In contrast to *A. platanoides*, bark samples of *C. sativa* contained substantial amounts of SQTs (Özgenç et al. 2017), which were not detected constitutively in intact trunks in the present study.

Constitutive trunk emissions responded to both abiotic and biotic stress, albeit to different extents. Relocation of two of the three studied *A. platanoides* trees to the quarantine facility, accompanied by elevated temperature and light intensity, resulted in increased emissions rates for most compounds. Temperature, artificial lighting, and elevated light intensity are all known to influence VOC release and biosynthesis (Loreto and Schnitzler 2010).

MTs exhibited the strongest response, where emissions of α-pinene, β-pinene, and 3-carene increased more markedly following relocation than after ALB infestation of one of the trees. This finding is notable for 3-carene, previously described as ALB-specific (Vermeeren et al. 2025), but is consistent with its detection in mechanically damaged maples (Makarow et al. 2020), suggesting that enzymatic pathways for their production were induced more considerably by abiotic stress than biotic stress.

Aldehyde emissions exhibited weaker stress responses than MTs but followed similar trends. Most aldehydes increased moderately under abiotic stress, consistent with the decline observed during initial acclimation and the subsequent rise after relocation. Positive correlations between light intensity and emissions of saturated C_6_–C_10_ aldehydes have been reported for *A. negundo* leaves (Hu et al. 2009), and temperature-dependent increases have been observed in apple trees (Vallat et al. 2005). In contrast, ALB infestation had little effect on aldehyde emissions. Although nonanal was stated to be reduced or actively inhibited by ALB infestation (Vermeeren et al. 2025), its emission remained stable or slightly increased at *Ap*I in this study. The discrepancy likely results from the normalization step prior to multivariate data analysis (Vermeeren et al. 2025), and emphasizes the importance of analyzing absolute temporal emission dynamics when interpreting up- or down-regulation. Hexanal, a GLV typically released at elevated levels from leaves after mechanical damage (Engelberth and Engelberth 2020), increased significantly upon relocation, but infestation-associated wounding did not further increase its emission, suggesting different regulations in trunks versus leaves.

Several constitutive trunk volatiles detected here are known to elicit electrophysiological or behavioral responses in ALB adults (Lyu et al. 2023), including nonanal, α-pinene, β-pinene, 3-carene, and limonene. Heptanal and nonanal additionally function as female pheromones, being attractive to both sexes of ALB adults (Xu and Teale 2021). These volatiles constituted a substantially higher proportion of trunk emissions (~ 26%, Table 2) than leaf emissions (~ 3%, (Zhang et al. 2008)), suggesting that not only leaf-derived but also trunk-derived volatiles may influence host selection preferences of ALB adults. Abiotic stress further increased the relative contribution of MTs, potentially enhancing host attractiveness. This is consistent with reports that drought-stressed trees are more attractive to ALB than healthy trees, resulting in more severe destruction of infestation sites (Wang et al. 2023), and parallel findings for other longhorned beetles such as the Japanese pine sawyer, *Monochamus alternatus*, which preferred stressed *Pinus massoniana* over healthy trees (Fan et al. 2007).

Compounds that are attractive to the ALB are also known to be attractive to its natural enemies *Dastarcus helophoroides* and potentially to hymenopteran wasps. Nonanal, α-pinene, 3-carene, and limonene attract the larval-pupae parasitoid *D. helophoroides* (Ren et al. 2017), and although behavioral studies on hymenopteran wasps are missing, studies on odorant-binding proteins (OBPs) in antennae from *Sclerodermus guani* revealed several volatiles including α-pinene, β-pinene, and 3-carene with high binding affinities to OBPs (Yi et al. 2018; Huang et al. 2023). Interestingly, OBPs showed complementary binding affinities, with some OBPs showing high binding affinities to aldehydes and aliphatics, and others to terpenoids and OCVs (Huang et al. 2023). These findings match with the dominating compound classes of constitutive and herbivore-induced plant volatiles, respectively, underscoring the hypothesis that constitutive trunk emissions support the attractiveness of HIPVs to carnivorous enemies. However, it is worth mentioning that high binding affinity values do not necessarily mean that these volatiles provoke a behavioral response, as demonstrated for decanal and limonene (Yi et al. 2018).

In contrast to constitutive trunk volatiles, ALB infestation caused pronounced changes in emission strength and VOC composition of *Ap*I’s trunk (Fig. 2). To our knowledge, this study represents the first temporal characterization of VOC emissions from the trunks of a living tree infested with wood-boring insects. In the absence of directly comparable studies, comparisons were made with a study on Norway spruce logs (*Picea abies* L.) infested with bark beetles (*Ips typographus* L.) (Du et al. 2025) and with studies on deciduous trees infested with foliar herbivores (Schaub et al. 2010; Maja et al. 2014; Giacomuzzi et al. 2016). When leaves become infested with insects, a burst release of GLVs usually correlating with feeding activity is observed, followed by delayed emission of terpenoids (Schaub et al. 2010; Maja et al. 2014; Giacomuzzi et al. 2016). For example, hybrid aspen (*P. tremula L.* × *P. tremuloides Michx.*) infested with Autumnal moth larvae (*Epirrita Autumnata*) showed terpene emission within 16 h (Schaub et al. 2010) and apple trees infested with apple brown tortrix *Pandemis heparana* larvae showed terpene emission starting to increase substantially on the second day after infestation (Giacomuzzi et al. 2016).

In contrast to foliar herbivory, no burst release of GLVs was observed from *Ap*I following ALB infestation, which suggests that GLVs may play a limited role in trunk-based herbivory. This hypothesis is supported by findings from bark beetle infested Norway spruce logs, which likewise did not emit GLVs (Du et al. 2025), indicating that tissue specificity does not only affect constitutive emissions but also HIPV emissions. Such distinct terpenoid blends for trunk vs. leaves have already been hypothesized by Courtois et al*.* based on the differences in terpenoids profiles of bark and leave samples of tropical tree species (Courtois et al. 2012).

Instead, terpenoids dominated the HIPV response of *Ap*I’s trunk to ALB infestation. MTs increased most strongly during the early stages of infestation and dominated emissions during oviposition, whereas SQTs became predominant once larvae probably began feeding on inner trunk tissue. It is worth noting that developmental times were estimated based on literature and are therefore subject to uncertainty. Beyond potential variability attributable to the nutrient medium (*i.e.*, host tree versus artificial diet), temperature represents a key determinant of developmental times of ALB eggs (Keena 2006) and larvae (Keena and Moore 2010). Uncertainty arising from temperature fluctuations within the quarantine facility was therefore represented as dashed triangles in Fig. 1.

The delayed dominance of SQTs relative to MTs mirrors observations from bark beetle infested Norway spruce logs, where the first emission peak was dominated by MTs and the second by SQTs, aromatics and oxygenated MTs (Du et al. 2025). Similar delays have also been reported for green apple aphid-infested (*Aphis pomi*) apple trees (Badra et al. 2021), although on much shorter timescales. However, foliar herbivory systems exhibit largely simultaneous increases in MT and SQT emissions (Schaub et al. 2010; Maja et al. 2014; Giacomuzzi et al. 2016).

The shift from MT- to SQT-dominated emissions may be linked to the nature of tissue disruption during infestation. Oviposition involves localized damage to bark and cambium (Meng et al. 2015; Lyu et al. 2023), whereas larvae feed primarily on phloem and xylem tissue (Lyu et al. 2023). Data on volatiles emitted from disrupted trunk tissue are not available; however, comparisons with maple syrup headspace analysis indicate that most HIPVs are not simply released from xylem sap (Sabik et al. 2010). Instead, their emission likely reflects induced biosynthesis in surrounding tissues. This interpretation is consistent with reports of terpenoids found in bark (Özgenç et al. 2017), phloem and xylem (Li et al. 2017) of other tree species.

An additional explanation for the observed temporal patterns is a shift from constitutive to induced defense strategies. As suggested for bark beetle-infested spruce logs, trees may first response by constitutive defense against herbivory, followed by induced defense under ongoing infestation (Du et al. 2025). This hypothesis aligns with our observation that most herbivore-induced MTs were already detectable prior to infestation, whereas SQTs largely appeared de novo. However, oviposition itself is known to induce specific volatile responses (Hilker and Fatouros 2015, 2016), indicating that it may be appropriate to distinguish between oviposition-induced and larval-induced volatiles.

Plant responses to egg deposition are increasingly recognized as distinct from responses to larval feeding (Hilker and Fatouros 2015, 2016). While direct defenses against egg deposition on leaves, such as cell death or the growth of neoplastic tissue to cast off or crush eggs (Hilker and Fatouros 2015), are less applicable to bark tissue, other mechanisms – including sap exudation and production of ovicidal compounds – may become relevant. Sap exudation, observed at *Ap*I in this study, is well documented for ALB oviposition (Meng et al. 2015), and poplars have been shown to kill ALB eggs by filling oviposition pits with plant fluids (Wang et al. 2023). In addition, MTs emitted at high concentration have been shown to exert physiological toxicity toward bark and pine beetles (Seybold et al. 2006; Reid et al. 2017). Therefore, the predominance of MTs during oviposition may represent a direct defensive strategy targeting eggs. As ALB females lay eggs over several weeks (Keena 2006), this plant response would explain the increase in MT emissions during oviposition, a correlation that has been reported for bark and wood-boring beetles (Amin et al. 2012; Zheng et al. 2021). Beside responses targeting the egg itself, egg-mediated plant defenses can be induced that affect the impending larval herbivory (Hilker and Fatouros 2016). The double-peak emission patterns observed for several MTs (Fig. 4 d) further suggest that these compounds may also target early larval stages. The higher first peak coinciding with the estimated start of egg hatching may indicate a primary defense against eggs, whereas the lower second peak coinciding with the estimated period of early xylem feeding may reflect either egg mortality or a shift toward indirect defense by attracting natural enemies. Volatiles emitted during oviposition have been shown to attract not only egg parasitoids but also parasitoids of later developmental stages, even before larvae hatch (Hilker and Fatouros 2015, 2016).

Consistent with this interpretation, many MTs identified here are known as HIPVs in coniferous and deciduous trees infested by bark- and wood-boring beetles. Although the composition of MTs identified in this study is only partially consistent with trunk emissions from ALB-infested *Acer* (spp.) (Makarow et al. 2020), most MTs have been reported to be leaf emissions from *Acer* (spp.) (Zhang et al. 2008; Wang et al. 2016; Ren et al. 2018). Furthermore, tricyclene, camphene, sabinene, β-pinene, myrcene, p-cymene, and γ-terpinene are known as HIPVs emitted from trunks of conifers upon infestation with bark beetles (Amin et al. 2013) or wood-boring beetles (Zheng et al. 2021), respectively. However, volatile profiles emitted from plants infested by wood-boring beetles (Cerambycidae) can differ from those with bark beetles (Scolytidae) not only because of the beetle’s species but also because of the potential introduction of fungi. Bark beetles are well-known vectors for symbiotic fungi that help the beetles colonize the host tree (Zhao et al. 2019) and the ALB can also introduce symbiotic gut fungi (Liu et al. 2025). The symbiotic presence of fungi can have notable impact on the emitted volatile blend as plants can also respond to pathogens (Kaur et al. 2022).

Herbivore-induced MTs, in contrast to constitutive MTs, showed greater variability in their effects on the attacking herbivore ALB and its parasitoid enemies *D. helophoroides* and *Sclerodermus* (spp.). Electrophysiological or behavioral data covering both the herbivore and its enemies are scarce; currently, such data is only available for limonene, which is attractive to ALB and *D. helophoroides* but does not elicit a specific behavioral response in *Sclerodermus* sp. (Yi et al. 2018), despite high binding affinities to OBPs of *S. guani* (Huang et al. 2023). For ALB and *S. guani*, camphene and β-myrcene also show high binding affinity in antennae of *S. guani* (Huang et al. 2023), with camphene being attractive and β-myrcene deterrent to ALB (Lyu et al. 2023). Moreover, although behavioral data for ALB and its parasitoids are lacking, DMNT it is widely recognized as a bifunctional semiochemical that repels herbivores while attracting natural enemies (Hatano et al. 2015).

From the perspective of the attacking herbivore, the relative contribution of attractive MTs (16%, including camphene, β-pinene, and limonene) exceeded that of repellent MTs (8%, including β-myrcene and β-ocimene). Notably, attractive and deterrent MTs exhibited different temporal dynamics. The deterrent β-ocimene did not correlate with any other herbivore-induced MT emitted from *Ap*I and was the earliest to increase, with elevated emission restricted to the oviposition period, suggesting a primary role in deterring further herbivores. In contrast, the temporal dynamics of the attractants camphene and β-pinene, closely resembled each other (Fig. 4 b), with limonene also showing moderately correlated dynamics characterized by transient emission peaks on individual days. Tricyclene displayed emission dynamics comparable to camphene and β-pinene, indicating a potential attractant function for ALB as well, although behavioral confirmation is currently lacking.

In contrast to the ALB, the proportion of MTs attractive to its natural enemies was markedly higher and dominated the herbivore-induced MT blend (59%, including camphene, β-myrcene, p-cymene, limonene, and γ-terpinene). In particular, p-cymene and γ-terpinene, contributing on average 40% to herbivore-induced MT emissions of *Ap*I, are known attractants for *D. helophoroides* (Yi et al. 2023). Because *D. helophoroides* is a larval-pupal parasitoid, the emission of attractant volatiles already during oviposition suggests that egg-induced volatiles can recruit not only egg parasitoids but also parasitoids targeting later developmental stages. Therefore, egg-mediated defensive functions described for foliar herbivory systems (Hilker and Fatouros 2015, 2016) also appear applicable to trunk-based herbivory by wood-boring insects.

Correlated temporal emission patterns were observed for p-cymene, γ-terpinene, and β-myrcene, indicating closely related upregulated biosynthesis. Sabinene and α-thujene showed quite similar emission patterns, suggesting that they may also contribute to parasitoid attraction, although behavioral data for these compounds are currently unavailable. Interestingly, grouping of MTs based on Kendall’s τ broadly corresponded to their behavioral classification as either parasitoid-attractive or ALB-attractive (Fig. 4). Moreover, elevated emissions of parasitoid-attracting MTs appeared to coincide with reduced emissions of ALB-attracting MTs, indicating potentially counteracting regulatory mechanisms. However, no significant negative correlation was detected and, given the limited behavioral data available for both ALB and its natural enemies, this pattern may also arise by chance.

Beyond parasitoids, MTs can also mediate attraction of avian predators. For example, emissions of β-ocimene, linalool, and DMNT were positively correlated with bird predation rates on artificial larvae placed on mountain birch (*Betula pubescens* ssp. *czerepanovii*) infested with caterpillars of the autumnal moth (*Epirrita autumnata*) (Mäntylä et al. 2008). β-Ocimene and DMNT were also detected at *Ap*I in this study, with DMNT being among the three most abundant MTs, suggesting an additional role in attracting higher trophic levels.

Collectively, these findings indicate that herbivore-induced MTs emitted from *Ap*I can contribute to indirect defense by both deterring additional herbivory and recruiting natural enemies. However, based on available behavioral evidence, the defense strategy appears to be dominated by attraction of natural enemies rather than by repellence of further herbivores or reduction of host attractiveness.

Sesquiterpenoid (SQT) emissions from *Ap*I peaked during the estimated period of larval feeding on xylem tissue (Fig. 2 c). In contrast to MTs, SQT emission patterns occurred later, were less variable, and showed greater similarity among compounds. SQTs are well recognized as key components of plant defense against both chewing and piercing-sucking herbivores (Leitner et al. 2005). Strong increases in SQT emissions have been reported from numerous infested plant species, including apple foliage, where herbivory resulted in 10 to 100-fold higher SQT emissions compared to intact foliage (Giacomuzzi et al. 2016).

The delayed emission maximum of SQTs observed in this study supports their primary role as indirect defense mechanism. For instance, SQTs are major parts in insect-induced volatiles emitted from maize and rice plants (Zhuang et al. 2012), to which the parasitic wasp *Cotesia marginiventris* (Cresson) was attracted to (Fontana et al. 2011). Nevertheless, high concentrations of zingiberene in tomato plants have shown to reduce oviposition and increase mortality of the South American corn rootworm *Diabrotica speciosa*, indicating that SQTs can also exert direct defensive effects (Nardi et al. 2024).

The SQTs dominating *Ap*I emission (cyclosativene, α-longipinene, α-copaene, zingiberene, and α-curcumene) were consistent with previous reports from ALB-infested maples (Makarow et al. 2020). Notably, several SQTs identified in this study – including α-longipinene, α-copaene, β-caryophyllene, and α-bergamotene – have also been reported to be contained in genital extracts of female ALB adults, with α-longipinene being predominant (Xu et al. 2020). This may explain the slightly different emission dynamic of α-longipinene compared to other SQTs observed at *Ap*I. Oviposition could introduce genital extracts of female adults, potentially contributing to the initial emission peak of α-longipinene (Fig. 5). However, as overall SQT emission peaked several weeks after oviposition – when adult beetles were no longer present – and other SQTs from female genital extracts were not detected at *Ap*I, emissions are likely dominated by plant-derived sources.

Despite their prevalence, behavioral and electrophysiological data for SQTs remain limited. β-Caryophyllene can be both attractive to ALB beetles when combined with host volatiles and deterrent when not (Lyu et al. 2023). Towards *D. helophoroides*, it elicited a strong repellant behavior (Ren et al. 2017), while other parasitoid wasps have shown to be attracted to β-caryophyllene (Köllner et al. 2008), and many OBPs in *S. guani* showed high binding affinities to β-caryophyllene and longifolene (Huang et al. 2023). Longifolene was also emitted from *Ap*I but was detected in ≤ 50% of measurements following infestation (Table S1). α-Longipinene has been shown to attract ALB beetles, with increasing attractiveness in the presence of host volatiles (Xu et al. 2020), as well as *S. guani*, despite low OBP binding affinities (Yi et al. 2018). In addition, α-copaene and α-bergamotene elicited electroantennographic (EAG) responses in antennae of male ALB adults (Xu et al. 2020) but behavioral data are missing. Considering the varying behavioral response in ALB beetles, the repellent responses observed for *D. helophoroides* towards isolated SQTs may not persist in natural blends. Reactions to a combination rather than individual compounds has been shown for virgin female wasps (*Chelonus insularis*), parasitizing eggs and larvae of the fall armyworm *Spodoptera frugiperda* (Ortiz-Carreon et al. 2019), and other parasitoids that frequently respond more strongly to blends containing both constitutive and herbivore-induced SQTs (Fontana et al. 2011). These findings support out hypothesis that constitutive volatiles should be considered along with HIPVs when interpreting ecological interactions.

As SQTs dominated VOC emissions during the estimated period of larval feeding, they represent promising candidates as chemical markers for ALB detection, particularly after adult beetles are no longer present. Makarow et al*.* already reported cyclosativene, α-longipinene, and α-copaene to give a strong hint to an ALB-infestation (Makarow et al. 2020). Multivariate data analysis on the same data based on pair-wise comparison of ALB-infested trees with healthy trees and larvae of other wood-boring insects such as goat moth *Cossus cossus* and poplar long-horned beetle *Saperda carcharias*, could not confirm these three SQTs to be specific, but zingiberene was stated as ALB-specific (Vermeeren et al. 2025). Nonetheless, persistent co-occurrence, stable relative ratios, and prevalence of cyclosativene, α-longipinene, α-copaene, zingiberene, and α-curcumene over several weeks have not been reported for other herbivores. In other systems, only subsets of these SQTs have been detected in combination: α-copaene and cyclosativene were emitted from *Medicago truncatula* after feeding by the cotton leafworm *Spodoptera littoralis* (Leitner et al. 2005); α-copaene and α-longipinene from maize plants infested with the fall armyworm *Spodoptera frugiperda* (Ortiz-Carreon et al. 2019) and from Norway spruce (*Picea abies* L.) infested with the bark beetle *Ips typographus* L. (Du et al. 2025); cyclosativene and α-longipinene were found in the xylem of pine trees (*Pinus massoniana*) infested with stem-boring *Monochamus alternatus* larvae (Li et al. 2017); zingiberene was reported to be emitted along with α-copaene from potato (*Solanum tuberosum*) plants, but only zingiberene was significantly higher in response to herbivory by *Spodoptera exigua* (Martín-Cacheda et al. 2023); and zingiberene along with α-curcumene was emitted from rice (*Oryza sativa*) plants upon herbivory of the stink bug (*Tibraca limbativentris*) (Melo Machado et al. 2014) and from potato (*Solanum tuberosum*) plants infested with the chewing herbivore Colorado Potato Beetle (*Leptinotarsa decemlineata* Say) or the piercing-sucking Green Peach Aphid (*Myzus persicae* Sulzer), respectively (Gosset et al. 2009). Therefore, targeting characteristic SQT blends rather than individual compounds appears to be a more robust strategy for identifying ALB infestation.

In addition to terpenoids, nitrogen- (NCVs) and some oxygen-containing volatiles (OCVs) were identified as HIPVs, none of which have previously been reported for ALB-infested trees. Their absence from earlier studies may reflect analytical limitations, such as stringent database match thresholds (Makarow et al. 2020). Although identification of several NCVs remained tentative due to limited database coverage, their molecular formulas could partly be confirmed by PTR–TOF measurements (Braun et al. 2025), and literature LRIs were available for some compounds.

Tentatively identified NCVs were largely exclusive to *Ap*I and were predominantly emitted during the first 13–14 weeks after infestation, suggesting a close association with herbivore activity. Although NCVs are less well studied than terpenoids, increasing evidence indicates that nitrogenous HIPVs can play important roles in plant–insect interactions. For example, studies on *Populus* (spp.) revealed cyanides, nitriles, nitro compounds, and several aldoximes to be emitted upon herbivory by gypsy moth caterpillars (*Lymantria dispar*), which can act as attractants to parasitoid braconid wasps (*Glyptapantheles liparidis*) (Irmisch et al. 2013). In particular, oximes – formed from amino acids catalyzed by cytochrome P450 monooxygenases from the CYP79 family and further convertible to nitriles – have been emphasized as underexplored components of plant defense (Sørensen et al. 2018; Twidle et al. 2022). As such, oximes are reported to potentially act as possible key attractants for parasitoids (Aljbory and Chen 2018), particularly for hymenopteran wasps (Wei and Kang 2006). Although comparable behavioral or electrophysiological data are lacking for ALB parasitoids specifically, the consistent and infestation-specific occurrence of tentatively identified NCVs in this study suggests a potential role in indirect defense that warrants further investigation.

The OCVs classified as HIPVs were less specific than terpenoids or NCVs and were also detected, albeit less frequently, in noninfested maples. These compounds likely originated from xylem sap exudation following oviposition-related wounding or as part of direct egg-defense responses. Ethanol, for example, has been shown to be transported through the xylem of *Populus tremula* (Portillo-Estrada and Niinemets 2018), and 2-butanone, 2-methyl-butanal, and acetic acid have been detected in the headspace of maple syrup (Sabik et al. 2010). Notably, acetic acid and 2-methyl-1-butanol are commonly used as attractants in commercial lures marketed in North America for hymenopteran wasps (Landolt and Zhang 2016). Although tested species do not include known ALB parasitoids, these compounds may nonetheless contribute to the attraction of natural enemies in ALB-infested systems. Taken together, NCVs and selected OCVs represent a previously overlooked component of the HIPV response in trunk-based herbivory and may complement terpenoid-mediated indirect defense.

The adjacent maple tree (*Ap*N) also exhibited altered volatile emissions, although to a lower extent. Two counteracting processes may explain these changes. On the one hand, *Ap*N and *Ap*I were both exposed to stress caused by pruning, relocation, and the climatic conditions within the quarantine facility. Consequently, emission profiles may have changed, potentially affecting attractiveness and resistance to herbivore attack. However, compounds that increased comparably in both maples within the quarantine facility are not known to either enhance or reduce host attractiveness to ALB. Considering ALB’s natural enemies, benzaldehyde and styrene that increased comparably at *Ap*N and *Ap*I have shown high binding affinities to OBPs of *S. guani* (Huang et al. 2023), potentially facilitating the parasitoids finding stressed trees. On the other hand, *Ap*N may have perceived VOCs emitted from the infested tree, triggering the induction of defense-related genes and VOC emissions in the neighboring, non-damaged tree. Such responses could either reduce susceptibility to herbivory or enhance attraction of natural enemies (Bezerra et al. 2021).

*Ap*N emitted the three SQTs (*Z*)-α-bergamotene, α-santalene, and cuparene at higher rates than *Ap*I and *Ap*C, respectively (Fig. 7). Although electroantennographic or behavioral data for ALB and its enemies are lacking, these SQTs have been shown to exert adverse effects on other herbivores. For instance, α-santalene and α-bergamotene negatively affected the performance and feeding of potato aphid (*Macrosiphum euphorbiae*) larvae on artificial diets and were reported to repel *M. euphorbiae* alatae (Wang et al. 2020). While effects of cuparene on phytophagous insects have not been reported, it is a major constituent of *Juniperus chinensis* wood oil, which is used as a repellent against ticks (Acari: Ixodidae) (Carroll et al. 2011). Thus, the induced emission of these SQTs may reduce the attractiveness of *Ap*N to ALB herbivory.

In addition to the SQTs, two alcohols (1-butanol and 1-penten-3—ol) were emitted at increased rates from *Ap*N associated with opposing effects on ALB. The attractant 1-butanol, previously reported to increase in drought-stressed *Acer negundo* leaves (Jin et al. 2004), was primarily emitted during the early stage of infestation of the adjacent *Ap*I. In contrast, the repellant 1-penten-3—ol was emitted at higher rates from *Ap*N when herbivory at *Ap*I was already advanced and emissions from *Ap*I had begun to decline. *Ap*N also released increased amounts of 2,4-dimethyl-1-heptene. Its role as a semiochemical remains unclear (Huang et al. 2023), and studies involving ALB, its natural enemies, or other hymenopteran insects are lacking. Nevertheless, 2,4-dimethyl-1-heptene has previously been identified as a strong indicator of ALB infestation (Makarow et al. 2020) and its specificity was confirmed by multivariate analyses (Vermeeren et al. 2025). In the present study, this compound did not appear as HIPV but as a volatile emitted from the adjacent maple. This observation does not necessarily contradict earlier findings, as trees may have been exposed to nearby infestations prior to becoming infested themselves. As such, priming effects have also been reported for different parts of the same plant (Dicke 2015).

Overall, it remains unclear whether the observed changes in the *Ap*N volatile emissions were driven by proximity to infestation or by other stress factors to which *Ap*I may have been less responsive due to ongoing herbivory. While many studies demonstrated that drought stress increases plant susceptibility to herbivores, the reverse scenario – how prior herbivory affects resistance to subsequent abiotic stress – remains poorly understood. Evidence from *A. artemisiifolia* plants suggests that drought tolerance can decline with increasing duration of reassociation with a specialist herbivore (Yin et al. 2023). If *Ap*N responded to neighboring infestation, the underlying signaling cues remain uncertain. Volatile compounds known to prime defenses in neighboring plants include salicylic acid, jasmonic acid, and their derivatives (Heil and Ton 2008). Of these, only methyl salicylate was detected at the trunk of *Ap*I, albeit in ≤ 30% of measurements. Notably, its emission persisted for approximately two weeks and coincided with the initial emission peak of several herbivore-induced MTs (Fig. 4 d). In this context, the delayed emission peaks of (*Z*)-α-bergamotene and 1-penten-3—ol may indicate a response to neighboring herbivory. Nonetheless, given the observed differences between trunk and leaf volatiles, priming signals beyond salicylic and jasmonic acid pathways may be relevant for wood-boring insects.

Concluding, this study provides a time-resolved characterization of volatile organic compound (VOC) emissions from the trunk of a living *Acer platanoides* infested by the Asian longhorned beetle (*Anoplophora glabripennis* Motschulsky). Using noninvasive sampling, we demonstrated that trunk-based herbivory triggered a prolonged and highly dynamic volatile response lasting several weeks, distinct from patterns reported for foliar herbivory. Constitutive trunk emissions were dominated by aldehydes and showed only limited induction by abiotic stress, while ALB infestation had negligible effects on their emission rates. In contrast, herbivore-induced emissions were terpenoid-rich and closely linked to the estimated stages of infestation: Monoterpenoids – dominated by p-cymene, (*E*)−4,8-Dimethylnona-1,3,7-triene, and γ-terpinene – prevailed during oviposition and early infestation, whereas sesquiterpenoids became dominant during the estimated period of larval feeding, particularly cyclosativene, α-longipinene, α-copaene, zingiberene, and α-curcumene. Many of these terpenoids are known or suspected semiochemicals mediating interactions with ALB and its natural enemies. The induced volatile blend contained compounds with both deterrent effects on herbivores and attractive effects on natural enemies, with the latter prevaling. Notably, volatiles associated with both egg-larval and larval-pupal parasitoid attraction were already emitted during oviposition, suggesting early recruitment of natural enemies as a potential key trunk defense strategy. In addition, previously unreported nitrogen- and oxygen-containing volatiles were detected that may further contribute to plant defense.

Overall, our findings highlight the importance of both blend composition and temporal emission dynamics in trunk-based plant defense and provide a mechanistic foundation for developing VOC-based strategies for ALB detection and monitoring. However, given the limitation to one tree per treatment due to quarantine constraints, future studies should validate these results by replication and include an extension of this approach to additional ALB host species to identify conserved and host-specific response patterns. Moreover, behavioral assays testing the attractiveness of complex trunk-emitted blends including combinations of herbivore-induced and constitutive volatiles, particularly sesquiterpenoids, will be essential to clarify their ecological functions and applied relevance.

## Electronic Supplementary Material

Below is the link to the electronic supplementary material.

Supplementary Material

## Data Availability

No datasets were generated or analysed during the current study.

## References

[CR1] Aljbory Z, Chen M (2018) Indirect plant defense against insect herbivores: a review. Insect Sci 25:2–23. 10.1111/1744-7917.12436 28035791 10.1111/1744-7917.12436

[CR2] Amin H, Atkins PT, Russo RS et al (2012) Effect of bark beetle infestation on secondary organic aerosol precursor emissions. Environ Sci Technol 46:5696–5703. 10.1021/es204205m 22545866 10.1021/es204205m

[CR3] Amin HS, Russo RS, Sive B et al (2013) Monoterpene emissions from bark beetle infested Engelmann spruce trees. Atmos Environ 72:130–133. 10.1016/j.atmosenv.2013.02.025

[CR4] Arimura G-i, Matsui K, Takabayashi J (2009) Chemical and molecular ecology of herbivore-induced plant volatiles: proximate factors and their ultimate functions. Plant Cell Physiol 50:911–923. 10.1093/pcp/pcp030 19246460 10.1093/pcp/pcp030

[CR5] Arnesen CH, Rosell F (2021) Pest detection dogs for wood boring longhorn beetles. Sci Rep. 10.1038/s41598-021-96450-0 34413443 10.1038/s41598-021-96450-0 PMC8376989

[CR6] Badra Z, Larsson Herrera S, Cappellin L et al (2021) Species-specific induction of plant volatiles by two aphid species in apple: real time measurement of plant emission and attraction of lacewings in the wind tunnel. J Chem Ecol 47:653–663. 10.1007/s10886-021-01288-5 34196858 10.1007/s10886-021-01288-5 PMC8346424

[CR7] Baldwin IT, Halitschke R, Paschold A et al (2006) Volatile signaling in plant-plant interactions: “talking trees” in the genomics era. Science 311:812–815. 10.1126/science.1118446 16469918 10.1126/science.1118446

[CR8] Bezerra RHS, Sousa-Souto L, Santana AEG, Ambrogi BG (2021) Indirect plant defenses: volatile organic compounds and extrafloral nectar. Arthropod-Plant Interact 15:467–489. 10.1007/s11829-021-09837-1

[CR9] Braun J, Engelhard C, Kaul P (2025) Optimized fast gas chromatography coupled with proton-transfer-reaction time-of-flight mass spectrometry for the selective near real-time analysis of herbivore-induced plant volatiles. J Chromatogr A. 10.1016/j.chroma.2025.466236 40737854 10.1016/j.chroma.2025.466236

[CR10] Carroll JF, Tabanca N, Kramer M et al (2011) Essential oils of *Cupressus funebris*, *Juniperus communis*, and *J. chinensis* (Cupressaceae) as repellents against ticks (Acari: Ixodidae) and mosquitoes (Diptera: Culicidae) and as toxicants against mosquitoes. J Vector Ecol 36:258–268. 10.1111/j.1948-7134.2011.00166.x 22129397 10.1111/j.1948-7134.2011.00166.x

[CR11] Chen M (2008) Inducible direct plant defense against insect herbivores: a review. Insect Sci 15:101–114. 10.1111/j.1744-7917.2008.00190.x

[CR107] Commission Implementing Regulation (EU) 2025/1952 of 29 September 2025 on measures to prevent the establishment and the spread within the Union territory of Anoplophora glabripennis (Motschulsky) and for the eradication and containment of that pest within certain demarcated areas and repealing Implementing Decision (EU) 2015/893. http://data.europa.eu/eli/reg_impl/2025/1952/oj

[CR12] Courtois EA, Baraloto C, Paine TC et al (2012) Differences in volatile terpene composition between the bark and leaves of tropical tree species. Phytochemistry 82:81–88. 10.1016/j.phytochem.2012.07.003 22863563 10.1016/j.phytochem.2012.07.003

[CR13] Dicke M (2015) Herbivore-induced plant volatiles as a rich source of information for arthropod predators: fundamental and applied aspects. J Indian Inst Sci 95:35–42

[CR14] Du B, Frühbrodt T, Delb H et al (2025) Emission patterns of volatile organic compounds from Norway spruce logs following bark beetle (*Ips typographus* L.) infestation. Tree Physiol. 10.1093/treephys/tpae152 39658196 10.1093/treephys/tpae152

[CR16] Dudareva N, Klempien A, Muhlemann JK, Kaplan I (2013) Biosynthesis, function and metabolic engineering of plant volatile organic compounds. New Phytol 198:16–32. 10.1111/nph.12145 23383981 10.1111/nph.12145

[CR15] Dudareva N, Negre F, Nagegowda DA, Orlova I (2006) Plant volatiles: recent advances and future perspectives. Crit Rev Plant Sci 25:417–440. 10.1080/07352680600899973

[CR17] Engelberth J, Engelberth M (2020) Variability in the capacity to produce damage-induced aldehyde green leaf volatiles among different plant species provides novel insights into biosynthetic diversity. Plants 9:213. 10.3390/plants9020213 32041302 10.3390/plants9020213 PMC7076675

[CR18] EPPO (2026) Anoplophora glabripennis. EPPO datasheets on pests recommended for regulation. In: Available online. https://gd.eppo.int (accessed 2026–03–29)

[CR19] Eyre D, Barbrook J (2021) The eradication of Asian longhorned beetle at Paddock Wood, UK. CABI Agric Biosci 2:12. 10.1186/s43170-021-00034-x

[CR20] Fan J, Sun J, Shi J (2007) Attraction of the Japanese pine sawyer, *Monochamus alternatus*, to volatiles from stressed host in China. Ann for Sci 64:67–71. 10.1051/forest:2006089

[CR21] FAO (2018) International Standards for Phytosanitary Measures. ISPM 15 Regulation of wood packaging material in international trade. Rome, Italy. https://openknowledge.fao.org/handle/20.500.14283/mb160e. Accessed 09 May 2026

[CR22] Fontana A, Held M, Fantaye CA et al (2011) Attractiveness of constitutive and herbivore-induced sesquiterpene blends of maize to the parasitic wasp *Cotesia marginiventris* (Cresson). J Chem Ecol 37:582–591. 10.1007/s10886-011-9967-7 21607717 10.1007/s10886-011-9967-7

[CR23] Gao G, Dai L, Gao J et al (2018) Volatile organic compound analysis of host and non-host poplars for *Trypophloeus klimeschi* (Coleoptera: Curculionidae: Ipinae). Russ J Plant Physiol 65:916–925. 10.1134/S1021443718060067

[CR24] Giacomuzzi V, Cappellin L, Khomenko I et al (2016) Emission of volatile compounds from apple plants infested with *Pandemis heparana* larvae, antennal response of conspecific adults, and preliminary field trial. J Chem Ecol 42:1265–1280. 10.1007/s10886-016-0794-8 27896554 10.1007/s10886-016-0794-8

[CR25] Gosset V, Harmel N, Göbel C et al (2009) Attacks by a piercing-sucking insect (*Myzus persicae* Sultzer) or a chewing insect (*Leptinotarsa decemlineata* Say) on potato plants (*Solanum tuberosum* L.) induce differential changes in volatile compound release and oxylipin synthesis. J Exp Bot. 10.1093/jxb/erp015 19221142 10.1093/jxb/erp015 PMC2657539

[CR27] Haack RA, Bauer LS, Gao R-T et al (2018) *Anoplophora glabripennis* within-tree distribution, seasonal development, and host suitability in China and Chicago. Great Lakes Entomol 39:169–183. 10.22543/0090-0222.2163

[CR26] Haack RA, Britton KO, Brockerhoff EG et al (2014) Effectiveness of the international phytosanitary standard ISPM No. 15 on reducing wood borer infestation rates in wood packaging material entering the United States. PLoS ONE 9:e96611. 10.1371/journal.pone.0096611 24827724 10.1371/journal.pone.0096611 PMC4020780

[CR28] Hatano E, Saveer AM, Borrero-Echeverry F et al (2015) A herbivore-induced plant volatile interferes with host plant and mate location in moths through suppression of olfactory signalling pathways. BMC Biol. 10.1186/s12915-015-0188-3 26377197 10.1186/s12915-015-0188-3 PMC4571119

[CR29] Heil M, Ton J (2008) Long-distance signalling in plant defence. Trends Plant Sci 13:264–272. 10.1016/j.tplants.2008.03.005 18487073 10.1016/j.tplants.2008.03.005

[CR30] Hilker M, Fatouros NE (2015) Plant responses to insect egg deposition. Annu Rev Entomol 60:493–515. 10.1146/annurev-ento-010814-020620 25341089 10.1146/annurev-ento-010814-020620

[CR31] Hilker M, Fatouros NE (2016) Resisting the onset of herbivore attack: plants perceive and respond to insect eggs. Curr Opin Plant Biol 32:9–16. 10.1016/j.pbi.2016.05.003 27267276 10.1016/j.pbi.2016.05.003

[CR32] Hoyer-Tomiczek U, Sauseng G, Hoch G (2016) Scent detection dogs for the Asian longhorn beetle, *Anoplophora glabripennis*. EPPO Bull 46:148–155. 10.1111/epp.12282

[CR35] Huang G, Liu Z, Gu S et al (2023) Identification and functional analysis of odorant-binding proteins of the parasitoid wasp *Scleroderma guani* reveal a chemosensory synergistic evolution with the host *Monochamus alternatus*. Int J Biol Macromol. 10.1016/j.ijbiomac.2023.126088 37532193 10.1016/j.ijbiomac.2023.126088

[CR34] Hu L, Meng |, Y |, Erb M (2018) Integration of two herbivore-induced plant volatiles results in synergistic effects on plant defence and resistance. Plant, Cell & Environment. 10.1111/pce.13443 10.1111/pce.13443 PMC6392123 30195252

[CR33] Hu Z, Shen Y, Su X (2009) Saturated aldehydes C6–C10 emitted from ashleaf maple (*Acer negundo* L.) leaves at different levels of light intensity, O2, and CO2. J Plant Biol 52:289–297. 10.1007/s12374-009-9035-9

[CR71] IPBES (2023) Summary for Policymakers of the Thematic Assessment Report on Invasive Alien Species and their Control of the Intergovernmental Science-Policy Platform on Biodiversity and Ecosystem Services. Roy, H. E., Pauchard, A., Stoett, P., Renard Truong, T., Bacher, S., Galil, B. S., Hulme, P. E., Ikeda, T., Sankaran, K. V., McGeoch, M. A., Meyerson, L. A., Nuñez, M. A., Ordonez, A., Rahlao, S. J., Schwindt, E., Seebens, H., Sheppard, A. W., and Vandvik, V. (eds.). IPBES secretariat, Bonn, Germany 10.5281/zenodo.7430692

[CR36] Irmisch S, McCormick AC, Boeckler AG et al (2013) Two herbivore-induced cytochrome P450 enzymes CYP79D6 and CYP79D7 catalyze the formation of volatile aldoximes involved in poplar defense. Plant Cell 25:4737–4754. 10.1105/tpc.113.118265 24220631 10.1105/tpc.113.118265 PMC3875747

[CR37] Jin Y, Li J, Li J et al (2004) Olfactory response of Anoplophora glabripennis to volatile compounds from ash-leaf maple (Acer ne- gundo) under drought stress. Scientia Silvae Sinicae 40:99–105

[CR38] Johnson CL, Coyle DR, Duan JJ et al (2025) A review of non-microbial biological control strategies against the Asian longhorned beetle (Coleoptera: Cerambycidae). Environ Entomol 54:679–690. 10.1093/ee/nvae116 39566077 10.1093/ee/nvae116 PMC12364626

[CR39] Kaur S, Samota MK, Choudhary M et al (2022) How do plants defend themselves against pathogens-biochemical mechanisms and genetic interventions. Physiol Mol Biol Plants 28:485–504. 10.1007/s12298-022-01146-y 35400890 10.1007/s12298-022-01146-y PMC8943088

[CR40] Keena MA (2006) Effects of temperature on *Anoplophora glabripennis* (Coleoptera: Cerambycidae) adult survival, reproduction, and egg hatch. Environ Entomol 35:912–921. 10.1603/0046-225X-35.4.912 10.1603/EN09369 22127184

[CR41] Keena MA, Moore PM (2010) Effects of temperature on *Anoplophora glabripennis* (Coleoptera: Cerambycidae) larvae and pupae. Environ Entomol 39:1323–1335. 10.1603/EN09369 22127184 10.1603/EN09369

[CR42] Köllner TG, Held M, Lenk C et al (2008) A maize ( *E* )-β-caryophyllene synthase implicated in indirect defense responses against herbivores is not expressed in most American maize varieties. Plant Cell 20:482–494. 10.1105/tpc.107.051672 18296628 10.1105/tpc.107.051672 PMC2276456

[CR43] Landolt P, Zhang Q-H (2016) Discovery and development of chemical attractants used to trap pestiferous social wasps (Hymenoptera: Vespidae). J Chem Ecol 42:655–665. 10.1007/s10886-016-0721-z 27435228 10.1007/s10886-016-0721-z

[CR44] Leitner M, Boland W, Mithöfer A (2005) Direct and indirect defences induced by piercing-sucking and chewing herbivores in *Medicago truncatula*. New Phytol 167:597–606. 10.1111/j.1469-8137.2005.01426.x 15998409 10.1111/j.1469-8137.2005.01426.x

[CR47] Li F, Zhang YL, Wang XY et al (2020) Discovery of parasitoids of *Anoplophora glabripennis* (Coleoptera: Cerambycidae) and their seasonal abundance in China using sentinel host eggs and larvae. J Econ Entomol 113:1656–1665. 10.1093/jee/toaa068 32300789 10.1093/jee/toaa068

[CR45] Li J, Jin Y, Luo Y et al (2003) Leaf volatiles from host tree *Acer negundo*: diurnal rhythm and behavior responses of *Anoplophora glabripennis* to volatiles in field. Acta Bot Sin 45:177–182

[CR48] Liu Q, Jia Y, Li Y et al (2025) Potential functions and transmission dynamics of fungi associated with *Anoplophora glabripennis* across different life stages, between sexes, and between habitats. Insects. 10.3390/insects16030273 40266779 10.3390/insects16030273 PMC11943397

[CR46] Li X, Dong G, Fang J et al (2017) Comparison of volatile organic compounds from uninfested and *Monochamus alternatus* Hope infested *Pinus massoniana* Lamb. Entomol Res 47:203–207. 10.1111/1748-5967.12209

[CR49] Loreto F, Schnitzler J-P (2010) Abiotic stresses and induced BVOCs. Trends Plant Sci 15:154–166. 10.1016/j.tplants.2009.12.006 20133178 10.1016/j.tplants.2009.12.006

[CR50] Lyu F, Hai X, Wang Z (2023) A review of the host plant location and recognition mechanisms of Asian longhorn beetle. Insects 14:292. 10.3390/insects14030292 36975977 10.3390/insects14030292 PMC10054519

[CR51] Maja MM, Kasurinen A, Yli-Pirila P et al (2014) Contrasting responses of silver birch VOC emissions to short- and long-term herbivory. Tree Physiol 34:241–252. 10.1093/treephys/tpt127 24627262 10.1093/treephys/tpt127

[CR52] Makarow R, Schäfer S, Albrecht S et al (2019) Investigation of volatile organic compounds emitted by *Anoplophora glabripennis* (Moschulsky) using thermal desorption and gas chromatography-mass spectrometry. Microchem J 146:142–148. 10.1016/j.microc.2018.12.036

[CR53] Makarow R, Schäfer S, Kaul P (2020) Identification of *Anoplophora glabripennis* (Moschulsky) by its emitted specific volatile organic compounds. Sci Rep 10:5194. 10.1038/s41598-020-61897-0 32251305 10.1038/s41598-020-61897-0 PMC7089994

[CR55] Martín-Cacheda L, Vázquez-González C, Rasmann S et al (2023) Plant genetic relatedness and volatile-mediated signalling between *Solanum tuberosum* plants in response to herbivory by *Spodoptera exigua*. Phytochemistry 206:113561. 10.1016/j.phytochem.2022.113561 36513136 10.1016/j.phytochem.2022.113561

[CR56] Melo Machado RC, Sant’Ana J, Blassioli-Moraes MC et al (2014) Herbivory-induced plant volatiles from *Oryza sativa* and their influence on chemotaxis behaviour of *Tibraca limbativentris* stal. (Hemiptera: Pentatomidae) and egg parasitoids. Bull Entomol Res 104:347–356. 10.1017/S0007485314000133 24622042 10.1017/S0007485314000133

[CR58] Meng PS, Hoover K, Keena MA (2015) Asian longhorned beetle (Coleoptera: Cerambycidae), an introduced pest of maple and other hardwood trees in North America and Europe. J Integr Pest Manag 6:. 10.1093/jipm/pmv003

[CR57] Men J, Zhao B, Cao DD et al (2019) Evaluating host location in three native *Sclerodermus* species and their ability to cause mortality in the wood borer *Aromia bungii* (Coleoptera: Cerambycidae) in laboratory. Biol Control 134:95–102. 10.1016/j.biocontrol.2019.04.007

[CR54] Mäntylä E, Alessio GA, Blande JD et al (2008) From plants to birds: higher avian predation rates in trees responding to insect herbivory. PLoS ONE 3:e2832. 10.1371/journal.pone.0002832 18665271 10.1371/journal.pone.0002832 PMC2475509

[CR59] Mumm R, Dicke M (2010) Variation in natural plant products and the attraction of bodyguards involved in indirect plant defense. Can J Zool 88:628–667. 10.1139/Z10-032

[CR60] Nardi C, Rech C, Ribeiro LK et al (2024) Tomato plants selected to high levels of zingiberene influence herbivory and fecundity of *Diabrotica speciosa*. Arthropod-Plant Interact 18:905–916. 10.1007/s11829-024-10091-4

[CR61] Nehme ME, Keena MA, Zhang A et al (2009) Attraction of *Anoplophora glabripennis* to male-produced pheromone and plant volatiles. Environ Entomol 38:1745–1755. 10.1603/022.038.0628 20021771 10.1603/022.038.0628

[CR62] Nehme ME, Keena MA, Zhang A et al (2010) Evaluating the use of male-produced pheromone components and plant volatiles in two trap designs to monitor *Anoplophora glabripennis*. Environ Entomol 39:169–176. 10.1603/EN09177 20146854 10.1603/EN09177

[CR63] Ortiz-Carreon FR, Rojas JC, Cisneros J, Malo EA (2019) Herbivore-induced volatiles from maize plants attract *Chelonus insularis*, an egg-larval parasitoid of the fall armyworm. J Chem Ecol 45:326–337. 10.1007/s10886-019-01051-x 30746603 10.1007/s10886-019-01051-x

[CR65] Portillo-Estrada M, Niinemets Ü (2018) Massive release of volatile organic compounds due to leaf midrib wounding in *Populus tremula*. Plant Ecol 219:1021–1028. 10.1007/s11258-018-0854-y 30395658 10.1007/s11258-018-0854-y PMC6047731

[CR66] Raguso RA (2008) Wake up and smell the roses: the ecology and evolution of floral scent. Annu Rev Ecol Evol Syst 39:549–569. 10.1146/annurev.ecolsys.38.091206.095601

[CR67] Reid ML, Sekhon JK, LaFramboise LM (2017) Toxicity of monoterpene structure, diversity and concentration to mountain pine beetles, *Dendroctonus ponderosae*: beetle traits matter more. J Chem Ecol 43:351–361. 10.1007/s10886-017-0824-1 28258318 10.1007/s10886-017-0824-1

[CR69] Ren H, Si F, Ye M et al (2018) Volatiles from *Acer truncatum* flowers. Am J Plant Sci 09:231–238. 10.4236/ajps.2018.92019

[CR68] Ren L, Balakrishnan K, Luo Y, Schütz S (2017) EAG response and behavioral orientation of *Dastarcus helophoroides* (Fairmaire) (Coleoptera: Bothrideridae) to synthetic host-associated volatiles. PLoS One. 10.1371/journal.pone.0190067 29267391 10.1371/journal.pone.0190067 PMC5739483

[CR70] Rissanen K, Vanhatalo A, Salmon Y et al (2020) Stem emissions of monoterpenes, acetaldehyde and methanol from Scots pine (*Pinus sylvestris* L.) affected by tree–water relations and cambial growth. Plant Cell Environ 43:1751–1765. 10.1111/pce.13778 32335919 10.1111/pce.13778

[CR72] Sabik H, Fortin J, Martin N (2010) Identification of volatile compounds in maple syrup using headspace solid-phase microextraction with gas chromatography-mass spectrometry. In: Quintin TJ (ed) Chromatography: Types. Nova Science Publishers Inc, Techniques and Methods, pp 417–427

[CR74] Schaub A, Blande JD, Graus M et al (2010) Real-time monitoring of herbivore induced volatile emissions in the field. Physiol Plant 138:123–133. 10.1111/j.1399-3054.2009.01322.x 20002328 10.1111/j.1399-3054.2009.01322.x

[CR75] Scholz M, Schütz S (2012) Trace analytical and electroantennographic examination of volatiles released by potential host trees and volatiles induced by *Anoplophora glabripennis* infestation. Forstschutz Aktuell 55:40–42

[CR76] Seybold SJ, Huber ADPW, Lee AJC et al (2006) Pine monoterpenes and pine bark beetles: a marriage of convenience for defense and chemical communication. Phytochem Rev 5:143–178. 10.1007/s11101-006-9002-8

[CR77] SmartLE, Aradottir GI, Bruce TJA (2014) Chapter 6 - Role of Semiochemicals in Integrated Pest Management. In: Abrol DP (ed) Integrated Pest Management. Academic Press, Cambridge, pp 93–109. 10.1016/B978-0-12-398529-3.00007-5

[CR78] Smith MT, Bancroft J, Tropp J (2002) Age-specific fecundity of *Anoplophora glabripennis* (Coleoptera: Cerambycidae) on three tree species infested in the United States. Environ Entomol 31:76–83. 10.1603/0046-225X-31.1.76

[CR73] Sánchez V, Keena MA (2013) Development of the teneral adult *Anoplophora glabripennis* (Coleoptera: Cerambycidae): time to initiate and completely bore out of maple wood. Environ Entomol 42:1–6. 10.1603/EN12225 23339780 10.1603/EN12225

[CR79] Sørensen M, Neilson EHJ, Møller BL (2018) Oximes: unrecognized chameleons in general and specialized plant metabolism. Mol Plant 11:95–117. 10.1016/j.molp.2017.12.014 29275165 10.1016/j.molp.2017.12.014

[CR80] Strutt A, Turner JA, Haack RA, Olson L (2013) Evaluating the impacts of an international phytosanitary standard for wood packaging material: global and United States trade implications. For Policy Econ 27:54–64. 10.1016/j.forpol.2012.11.003

[CR82] Twidle AM, Barker D, Pilkington LI et al (2022) Identification of herbivore-induced plant volatiles from selected *Rubus* species fed upon by raspberry bud moth (*Heterocrossa rubophaga*) larvae. Phytochemistry. 10.1016/j.phytochem.2022.113325 35843359 10.1016/j.phytochem.2022.113325

[CR83] Unsicker SB, Kunert G, Gershenzon J (2009) Protective perfumes: the role of vegetative volatiles in plant defense against herbivores. Curr Opin Plant Biol 12:479–485. 10.1016/j.pbi.2009.04.001 19467919 10.1016/j.pbi.2009.04.001

[CR84] Vallat A, Gu H, Dorn S (2005) How rainfall, relative humidity and temperature influence volatile emissions from apple trees in situ. Phytochemistry 66:1540–1550. 10.1016/j.phytochem.2005.04.038 15949824 10.1016/j.phytochem.2005.04.038

[CR85] van Den Dool H, Kratz PD (1963) A generalization of the retention index system including linear temperature programmed gas-liquid partition chromatography. J Chromatogr A 11:463–471. 10.1016/s0021-9673(01)80947-x 10.1016/s0021-9673(01)80947-x 14062605

[CR86] van der Gaag DJ, Loomans AJM (2014) Host plants of *Anoplophora glabripennis*, a review. EPPO Bull 44:518–528. 10.1111/epp.12151

[CR87] Vermeeren S, Witzler M, Makarow R et al (2025) Multivariate evaluation method for the detection of pest infestations on plants via VOC analysis using gas chromatography mass spectrometry. Sci Rep 15:25858. 10.1038/s41598-025-11607-5 40670585 10.1038/s41598-025-11607-5 PMC12267415

[CR88] Vickers CE, Gershenzon J, Lerdau MT, Loreto F (2009) A unified mechanism of action for volatile isoprenoids in plant abiotic stress. Nat Chem Biol 5:283–291. 10.1038/nchembio.158 19377454 10.1038/nchembio.158

[CR91] Wang F, Park YL, Gutensohn M (2020) Glandular trichome-derived sesquiterpenes of wild tomato accessions (*Solanum habrochaites*) affect aphid performance and feeding behavior. Phytochemistry. 10.1016/j.phytochem.2020.112532 33045464 10.1016/j.phytochem.2020.112532

[CR90] Wang L, Li C, Luo Y et al (2023) Current and future control of the wood-boring pest *Anoplophora glabripennis*. Insect Sci 30:1534–1551. 10.1111/1744-7917.13187 36944595 10.1111/1744-7917.13187

[CR89] Wang Q, Liu H, Wang B et al (2016) Component analysis of volatile organic compounds from branches and leaves in seven *Acer* species. Zhejiang Nonglin Daxue Xuebao 33:524–530

[CR92] War AR, Paulraj MG, Ahmad T et al (2012) Mechanisms of plant defense against insect herbivores. Plant Signal Behav 7:1306–1320. 10.4161/psb.21663 22895106 10.4161/psb.21663 PMC3493419

[CR93] Wei JN, Kang L (2006) Electrophysiological and behavioral responses of a parasitic wasp to plant volatiles induced by two leaf miner species. Chem Senses 31:467–477. 10.1093/chemse/bjj051 16621971 10.1093/chemse/bjj051

[CR94] Wei JR, Yang ZQ, Hao HL, Du JW (2008) (R)-(+)-limonene, kairomone for *Dastarcus helophoroides*, a natural enemy of longhorned beetles. Agric For Entomol 10:323–330. 10.1111/j.1461-9563.2008.00384.x

[CR95] Wickham JD, Xu Z, Teale SA (2012) Evidence for a female-produced, long range pheromone of *Anoplophora glabripennis* (Coleoptera: Cerambycidae). Insect Sci 19:355–371. 10.1111/j.1744-7917.2012.01504.x

[CR97] Xu T, Hansen L, Cha DH et al (2020) Identification of a female-produced pheromone in a destructive invasive species: Asian longhorn beetle, *Anoplophora glabripennis*. J Pest Sci 93(4):1321–1332. 10.1007/s10340-020-01229-3

[CR98] Xu T, Hansen L, Teale SA (2021) Mating and adult feeding behaviour influence pheromone production in female Asian longhorn beetle *Anoplophora glabripennis* (Coleoptera: Cerambycidae). Agric For Entomol 23:276–286. 10.1111/afe.12430

[CR96] Xu T, Teale SA (2021) Chemical ecology of the Asian longhorn beetle, *Anoplophora glabripennis*. J Chem Ecol 47:489–503. 10.1007/s10886-021-01280-z 34081236 10.1007/s10886-021-01280-z

[CR101] Yin W, Zhou L, Yang K et al (2023) Rapid evolutionary trade-offs between resistance to herbivory and tolerance to abiotic stress in an invasive plant. Ecol Lett 26:942–954. 10.1111/ele.14221 37078102 10.1111/ele.14221

[CR100] Yi S-C, Wu Y-H, Yang R-N et al (2023) A highly expressed antennae odorant-binding protein involved in recognition of herbivore-induced plant volatiles in *Dastarcus helophoroides*. Int J Mol Sci 24:3464. 10.3390/ijms24043464 36834874 10.3390/ijms24043464 PMC9962305

[CR99] Yi SY, Li DZ, Zhou CX et al (2018) Screening behaviorally active compounds based on fluorescence quenching in combination with binding mechanism analyses of SspOBP7, an odorant binding protein from *Sclerodermus sp.*. Int J Biol Macromol 107:2667–2678. 10.1016/j.ijbiomac.2017.10.149 29113892 10.1016/j.ijbiomac.2017.10.149

[CR64] Özgenç DS, Çelik G et al (2017) Comparative phytochemical analysis of volatile organic compounds by SPME-GC-FID/MS from six coniferous and nine deciduous tree bark species grown in Turkey. S Afr J Bot 113:23–28. 10.1016/j.sajb.2017.07.004

[CR103] Zhang F, Jin Y, Chen H, Wu X (2008) Selectivity mechanism of *Anoplophora glabripennis* on four different species of maples. Front Biol China 3:78–84. 10.1007/s11515-008-0006-1

[CR102] Zhang Q-H, Birgersson G, Zhu J et al (1999) Leaf volatiles from nonhost deciduous trees: variation by tree species, season and temperature, and electrophysiological activity in *Ips typographus*. J Chem Ecol 25:1923–1943

[CR104] Zhao T, Ganji S, Schiebe C et al (2019) Convergent evolution of semiochemicals across kingdoms: bark beetles and their fungal symbionts. ISME J 13:1535–1545. 10.1038/s41396-019-0370-7 30770902 10.1038/s41396-019-0370-7 PMC6776033

[CR105] Zheng C, Wang Z, Zhang J et al (2021) Discrimination of wood-boring beetles infested *Platycladus orientalis* plants by using gas chromatography-ion mobility spectrometry. Comput Electron Agric 180:105896. 10.1016/j.compag.2020.105896

[CR106] Zhuang X, Köllner TG, Zhao N et al (2012) Dynamic evolution of herbivore-induced sesquiterpene biosynthesis in sorghum and related grass crops. Plant J 69:70–80. 10.1111/j.1365-313X.2011.04771.x 21880075 10.1111/j.1365-313X.2011.04771.x

