# Supplementary material for: Emission Dynamics of Constitutive and Herbivore-induced Plant Volatiles from Norway Maple (Acer platanoides) Trunk Infested by the Asian Longhorned Beetle (Anoplophora glabripennis (Motschulsky))
Source: J Chem Ecol. 2026 May 21;52(3):46. doi: 10.1007/s10886-026-01718-2 (PMC13194219; doi:10.1007/s10886-026-01718-2)
Supplement: Supplementary file 1 — Supplementary Material [file 10886_2026_1718_MOESM1_ESM.docx]

# SUPPLEMENTARY INFORMATION





**Fig. S 1** Picture and schematic drawing of the ventilated in-house quarantine facility with two chambers, separated by metal grids (dotted line). The maple tree infested with ALB (*Ap*I) was situated in the right-hand chamber, while the similarly treated but noninfested maple tree (*Ap*N) was located in the left-hand chamber. A control tree located outside the quarantine facility was grown in pots indoors but has not been pruned


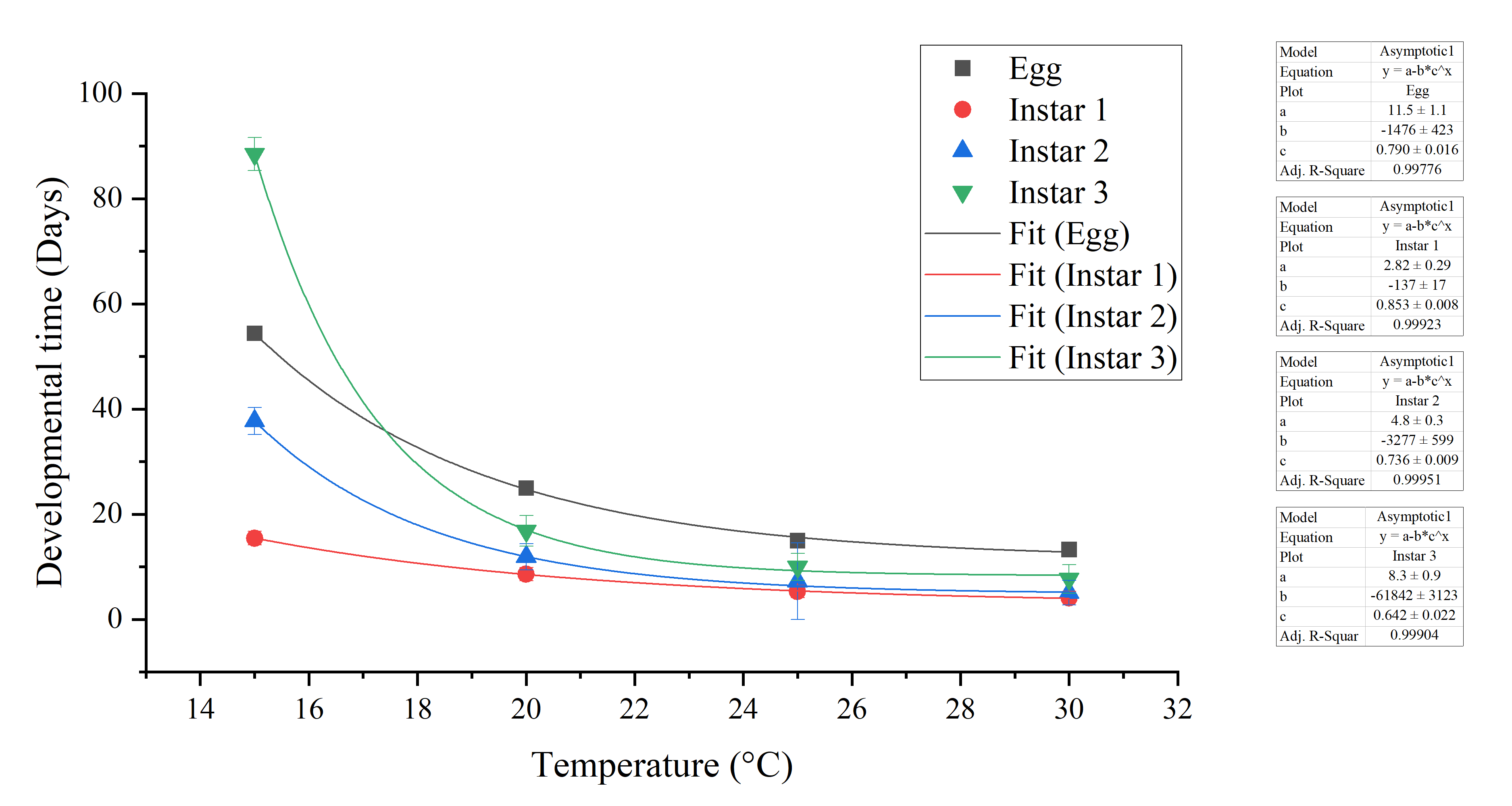


**Fig. S 2** Developmental times of ALB eggs until hatching and immature stages of ALB larvae at different temperatures, based on published data (Keena 2006; Keena and Moore 2010). Data of Keena *et al.* were fitted by the asymptotic regression model with 1^st^ parameterization from OriginLab, following the fitting function $y=a-b^{x}$. Adjusted R²-values were >0.997 for fits of all developmental stages.


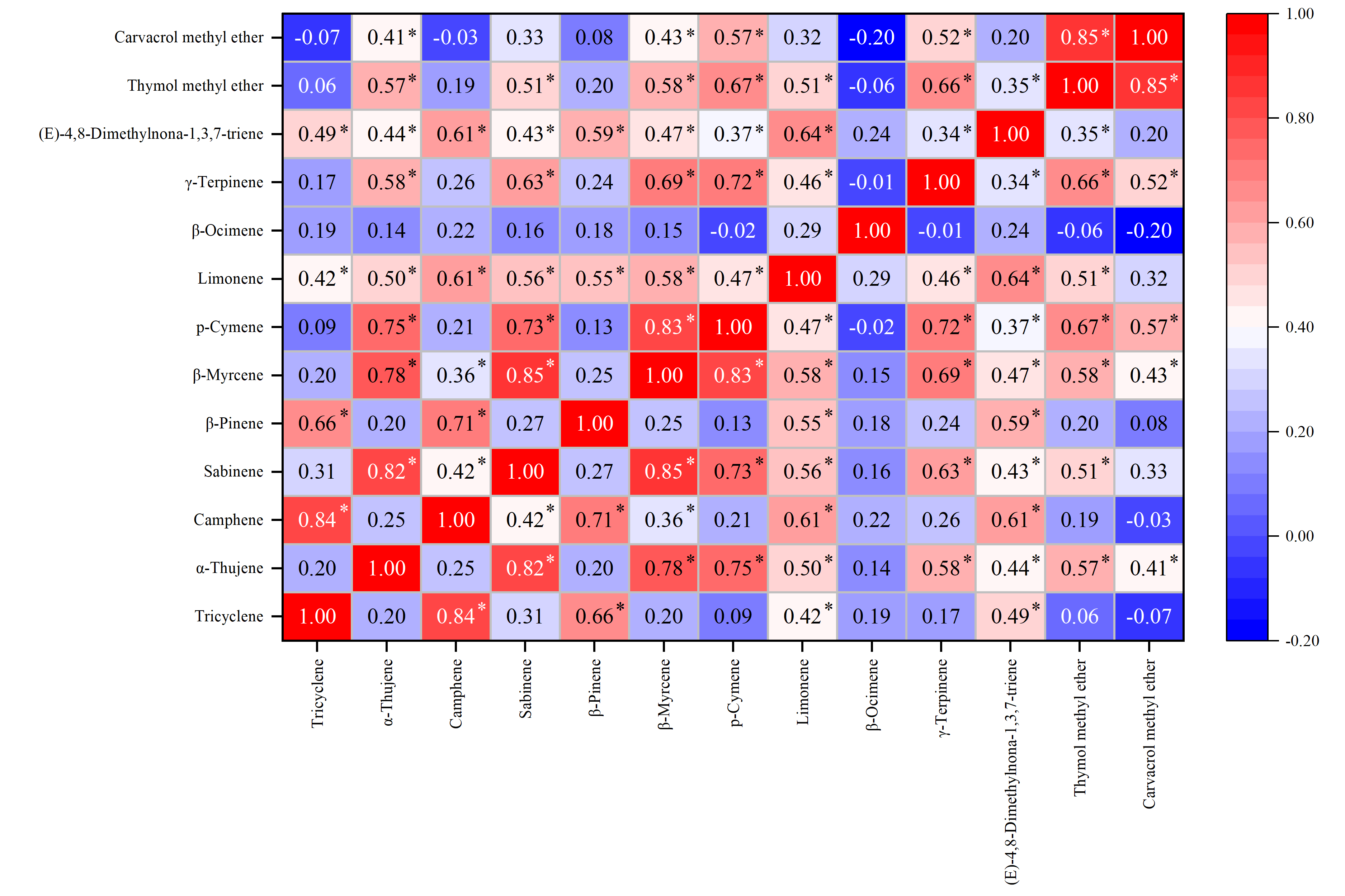


**Fig. S 3** Kendall’s tau correlation coefficients (τ) of monoterpenoids identified as HIPVs at the trunk of the ALB-infested *Ap*I, based on emission rates within the first 12 weeks after ALB exposure. Significant coefficients at *P* > 0.05 are marked with an asterisk (*)


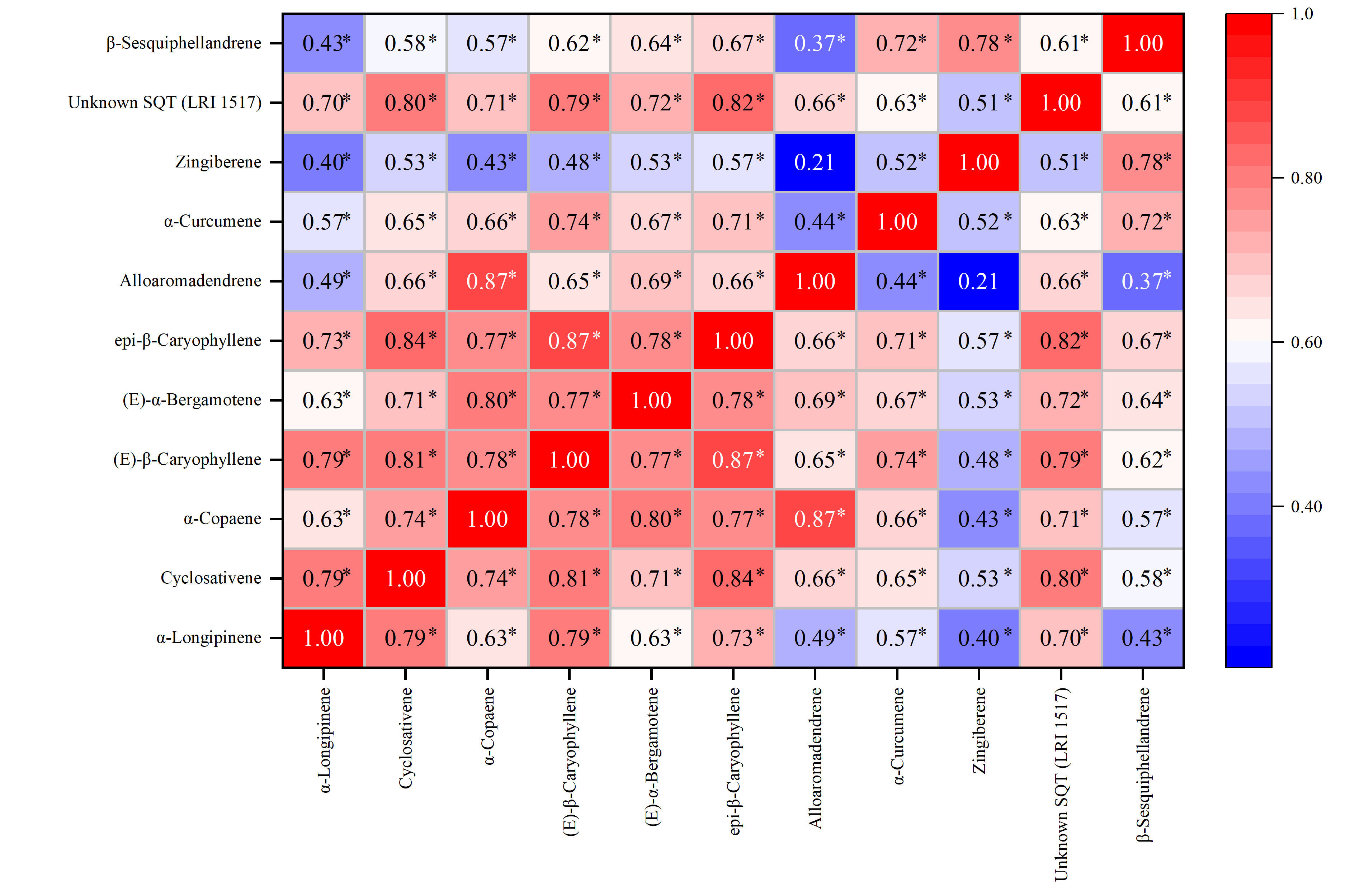


**Fig. S 4** Kendall’s tau correlation coefficients (τ) of sesquiterpenoids identified as HIPVs at the trunk of the ALB-infested *Ap*I, based on emission rates within the first 12 weeks after ALB exposure. Significant coefficients at *P* > 0.05 are marked with an asterisk (*)

**Table S 1** Herbivore-induced plant volatiles emitted from the trunk of a Norway maple (*Acer platanoides*) infested with the Asian longhorned beetle (ALB, *Anoplophora glabripennis* Motschulsky), detected in 30–50 % of the measurements within the first 24 weeks after ALB exposure. References comprise VOCs emitted from different parts of *Acer* (spp.) and with relevance to ALB, and *Sclerodermus* (spp.) as attractant (A), repellent (R), neutral (N) or – depending on their concentration – both (A/R). Data on *Sclerodermus* (spp.) were supplemented by volatiles which showed binding affinities (BA) to odorant-binding proteins in antennae

| LRI  exp. | MF | Compound name | CAS | ID ^a^ | NIST20 | | DF (%)  (N = 61) | Emission rate ratio ^b^ | | | References | | | | |
| --- | --- | --- | --- | --- | --- | --- | --- | --- | --- | --- | --- | --- | --- | --- | --- |
|  |  |  |  |  | SM | LRI lit. |  | Inter-tree | | Intra-tree | *Acer* (spp.) | | | Semiochemicals | |
|  |  |  |  |  |  |  |  | *Ap*I*/ Ap*N | *Ap*I*/ Ap*N | pre/post  ALB | Trunk | Canopy | Syrup | ALB | *Sclerodermus*  (spp.) |
|  |  | *Mono- and homoterpenoids* |  |  |  |  |  |  |  |  |  |  |  |  |  |
| 948 | C_10_H_18_ | β-Citronellene | 2436-90-0 | M, R | 88 (7) | 946 | 39 | *Ap*I | *Ap*I | ALB |  |  |  |  |  |
| 1020 | C_10_H_16_ | α-Terpinene | 99-86-5 | M, R | 87 (5) | 1017 | 48 | *Ap*I | *Ap*I | ALB |  |  |  |  |  |
| 1092 | C_10_H_16_ | Unknown | n.a. | n.a. | 77 (4) | n.a. | 41 | > | *Ap*I | > |  |  |  |  |  |
| 1094 | C_10_H_12_ | p-Cymenene | 1195-32-0 | M, R | 89 (3) | 1090 | 48 | > | *Ap*I | > |  |  |  |  |  |
| 1098 | C_11_H_18_ | (Z)-4,8-Dimethylnona-1,3,7-triene | n.a. | M, R | 81 (6) | 1097 | 33 | *Ap*I | *Ap*I | ALB |  |  |  |  |  |
| 1173 | C_10_H_18_O | Borneol | 507-70-0 | M, R, S | 81 (7) | 1167 | 31 | *Ap*I | *Ap*I | ALB |  |  |  |  |  |
| 1205 | C_10_H_12_O | Unknown | n.a. | n.a. | n.a. | n.a. | 33 | > | > | ALB |  |  |  |  |  |
| 1235 | C_11_H_16_O | Isothymol methyl ether | 31574-44-4 | M, R | 87 (3) | 1230 | 31 | *Ap*I | *Ap*I | ALB |  |  |  |  |  |
|  |  | *Sesquiterpenoids* |  |  |  |  |  |  |  |  |  |  |  |  |  |
| 1310 | C_15_H_24_ | Unknown | n.a. | n.a. | n.a. | n.a. | 33 | *Ap*I | *Ap*I | ALB |  |  |  |  |  |
| 1347 | C_15_H_24_ | Unknown | n.a. | n.a. | n.a. | n.a. | 36 | *Ap*I | > | ALB |  |  |  |  |  |
| 1367 | C_15_H_24_ | Unknown | n.a. | n.a. | n.a. | n.a. | 44 | *Ap*I | *Ap*I | ALB |  |  |  |  |  |
| 1396 | C_15_H_24_ | Sesquithujene | 58319-06-5 | M, R | 85 (6) | 1402 | 49 | *Ap*I | *Ap*I | > |  |  |  |  |  |
| 1409 | C_15_H_24_ | Cyperene | 2387-78-2 | M, R | 80 (5) | 1399 | 49 | *Ap*I | *Ap*I | ALB |  |  |  |  |  |
| 1418 | C_15_H_24_ | Longifolene | 475-20-7 | M, R, S | 84 (5) | 1406 | 49 | > | > | ALB | (Makarow et al. 2020) |  |  |  | BA (Huang et al. 2023) |
| 1421 | C_15_H_24_ | α-Gurjunene | 489-40-7 | M, R | 85 (4) | 1409 | 46 | *Ap*I | *Ap*I | ALB | (Makarow et al. 2020) |  |  |  |  |
| 1448 | C_15_H_24_ | Seychellene | 20085-93-2 | M, R | 84 (3) | 1459 | 46 | 5 | *Ap*I | ALB |  |  |  |  |  |
| 1455 | C_15_H_24_ | Valerena-4,7(11)-diene | 351222-66-7 | M, R | 77 (4) | 1460 | 46 | *Ap*I | *Ap*I | ALB |  |  |  |  |  |
| 1524 | C_15_H_24_ | α-Bulnesene | 3691-11-0 | M, R | 78 (4) | 1505 | 48 | *Ap*I | *Ap*I | ALB |  |  |  |  |  |
| 1656 | C_15_H_20_O | Unknown | n.a. | n.a. | n.a. | n.a. | 38 | *Ap*I | *Ap*I | ALB |  |  |  |  |  |
| 1765 | C_15_H_22_O | Xanthorrhizol | 30199-26-9 | M, R, S | 84 (6) | 1753 | 48 | *Ap*I | *Ap*I | ALB |  |  |  |  |  |
|  |  | *Nitrogen-containing compounds* |  |  |  |  |  |  |  |  |  |  |  |  |  |
| 709 | C_4_H_5_NS | Unknown | n.a. | n.a. | n.a. | n.a. | 43 | *Ap*I | *Ap*I | ALB |  |  |  |  |  |
| 805 | C_6_H_11_NO | Unkown oxime | n.a. | n.a. | n.a. | n.a. | 48 | *Ap*I | *Ap*I | ALB |  |  |  |  |  |
| 903 | C_5_H_11_NO_2_ | 1-Nitropentane | 628-05-7 | M, R | 92 (3) | 896 | 49 | *Ap*I | *Ap*I | ALB |  |  |  |  |  |
| 1493 | C_21_H_33_NO_3_ | Unknown | n.a. | n.a. | n.a. | n.a. | 39 | *Ap*I | *Ap*I | > |  |  |  |  |  |
|  |  | *Oxygen-containing compounds* |  |  |  |  |  |  |  |  |  |  |  |  |  |
| 620 | C_4_H_8_O_2_ | Ethyl Acetate | 141-78-6 | M, R, S | 88 (7) | 612 | 38 | 34 | 7 | 66.0 |  | (Zhang et al. 2008) |  | A (Lyu et al. 2023) | BA (Huang et al. 2023) |
| 732 | C_3_H_6_O_2_ | Propanoic acid | 79-09-4 | M, R | 96 (5) | 706 | 44 | > | > | > |  |  |  |  |  |
| 742 | C_5_H_12_O | 3-Methyl-1-butanol | 123-51-3 | M, R | 94 (4) | 736 | 31 | 4 | 4 | 2.7 |  |  |  |  |  |
| 756 | C_4_H_8_O_2_ | Acetoin | 513-86-0 | M, R, S | 89 (7) | 713 | 34 | 26 | > | ALB |  |  |  |  |  |
| 826 | C_4_H_10_O_2_ | 2,3-Butanediol | 513-85-9 | M, R, S | 86 (8) | 788 | 36 | > | > | ALB |  |  | (Sabik et al. 2010) |  | N (Huang et al. 2023) |
| 932 | C_4_H_6_O_2_ | Butyrolactone | 96-48-0 | M, R | 89 (7) | 916 | 34 | > | > | ALB |  |  |  |  |  |
| 1677 | C_17_H_32_ | 6,9-Heptadecadiene | 81265-03-4 | M, R | 87 (6) | 1667 | 43 | > | > | > |  |  |  |  |  |
| 1801 | C_16_H_32_O_2_ | n-Hexadecanoic acid | 57-10-3 | M, R | 93 (8) | 1968 | 49 | > | > | ALB |  |  |  |  |  |

LRI = linear retention index, MF = molecular formula, ID = identification methods, SM = spectral match, given as mean (standard deviation), DF = detection frequency, *Ap*I = ALB-infested, *Ap*N = neighboring to ALB-infestation, *Ap*C = control.

^a^ Identification methods: M = comparison of the mass spectrum with those contained in NIST20 Library, R = comparison of experimental LRI with literature LRI contained in NIST20 Library, S = Confirmation with analytical standards

^b^ For inter-tree comparison, ratio was calculated from component areas averaged over period 3 (after *Ap*I has been exposed to ALB), and for *Ap*I intra-tree comparison, ratio was calculated from component areas averaged over period 1–2 (N = 20) and 3 (N = 61), respectively. Only component areas of compounds that were detected in at least 20 % of measurements per sample type and period were used for calculation. Inter-tree ratios of compounds are marked with ‘*Ap*I‘ if they were exclusively detected at *Ap*I, and with ‘>’ if they appeared in less than 20 % of measurements of the noninfested maples. Intra-tree ratios are marked with ‘ALB’ if the respective compound appeared *de novo* after ALB exposure or in less than 20 % of measurements prior ALB exposure.

**Table S 2** Induced plant volatiles emitted from the trunk of a Norway maple (*Acer platanoides*) neighboring to an ALB-infested maple but separated from beetles by metal grid, detected in ≥ 30 % of measurements within the first 24 weeks after neighboring infestation. References comprise VOCs emitted from different parts of *Acer* (spp.) and with relevance to ALB and *Sclerodermus* (spp.) as attractant (A), repellent (R), or neutral (N)

| LRI  exp. | MF | Compound name | CAS | ID ^a^ | NIST20 | | DF (%)  (N = 63) | Emission rate ratio ^b^ | | | References | | | | |
| --- | --- | --- | --- | --- | --- | --- | --- | --- | --- | --- | --- | --- | --- | --- | --- |
|  |  |  |  |  | SM | LRI  lit. |  | Inter-tree | | Intra-tree | *Acer* (spp.) | | | Semiochemicals | |
|  |  |  |  |  |  |  |  | *Ap*I*/ Ap*N | *Ap*I*/ Ap*N | pre/post  ALB | Trunk | Canopy | Syrup | ALB | *Sclerodermus* (spp.) |
|  |  | *Sesquiterpenoids* |  |  |  |  |  |  |  |  |  |  |  |  |  |
| 1361 | C_15_H_24_ | α-Longipinene | 5989-08-2 | M, R, S | 93 (6) | 1353 | 86 | <1 | 2 | 4 | (Makarow et al. 2020) |  |  | A (Xu et al. 2020) |  |
| 1423 | C_15_H_24_ | (*Z*)-α-Bergamotene | 18252-46-5 | M, R | 94 (2) | 1415 | 94 | > | > | 5 |  |  |  | EAG (Xu et al. 2020) |  |
| 1429 | C_15_H_24_ | α-Santalene | 512-61-8 | M, R, S | 81 (5) | 1420 | 68 | > | ApN | ALB |  |  |  |  |  |
| 1444 | C_15_H_24_ | (*E*)-α-Bergamotene | 13474-59-4 | M, R, S | 92 (3) | 1435 | 83 | <1 | > | 6 |  |  |  | EAG (Xu et al. 2020) |  |
| 1491 | C_15_H_22_ | α-Curcumene | 644-30-4 | M, R, S | 91 (7) | 1483 | 73 | <1 | > | 2 | (Makarow et al. 2020) |  |  |  |  |
| 1520 | C_15_H_22_ | Cuparene | 16982-00-6 | M, R, S | 81 (6) | 1504 | 57 | ApN | > | > |  |  |  |  |  |
|  |  | *Ketones* |  |  |  |  |  |  |  |  |  |  |  |  |  |
| 698 | C_5_H_10_O | 2-Pentanone | 107-87-9 | M, R | 83 (4) | 685 | 49 | > | > | > |  |  |  |  |  |
| 896 | C_7_H_14_O | 2-Heptanone | 110-43-0 | M, R | 86 (5) | 891 | 54 | > | > | > |  |  |  |  |  |
| 960 | C_8_H_16_O | 6-Methyl-2-heptanone | 928-68-7 | M, R | 76 (3) | 956 | 35 | > | > | > |  | (Zhang et al. 2008) |  |  |  |
| 1096 | C_9_H_18_O | 2-Nonanone | 821-55-6 | M, R | 81 (5) | 1092 | 49 | > | > | ALB |  |  |  |  |  |
|  |  | *Hydrocarbons* |  |  |  |  |  |  |  |  |  |  |  |  |  |
| 622 | C_6_H_12_ | Methylcyclopentane | 96-37-7 | M, R | 87 (6) | 630 | 33 | > | > | > |  | (Zhang et al. 2008) |  |  |  |
| 821 | C_9_H_20_ | 2,4-Dimethylheptane | 2213-23-2 | M, R | 88 (5) | 821 | 49 | > | > | 2 |  |  |  |  |  |
| 841 | C_9_H_18_ | 2,4-Dimethyl-1-heptene | 19549-87-2 | M, R, S | 91 (6) | 836 | 44 | > | > | 7 | (Makarow et al. 2020) |  |  |  | N (Huang et al. 2023) |
| 1102 | C_12_H_16_ | 2,4-Dimethyldecane | 2801-84-5 | M, R | 81 (4) | 1106 | 38 | > | > | > |  |  |  |  |  |
| 1281 | C_13_H_28_ | Ethylundecane | 17312-58-2 | M, R | 83 (4) | 1260 | 40 | > | > | > |  |  |  |  |  |
|  |  | *Alcohols* |  |  |  |  |  |  |  |  |  |  |  |  |  |
| 674 | C_4_H_10_O | 1-Butanol | 71-36-3 | M, R, S | 91 (4) | 659 | 46 | > | > | ALB |  | (Li et al. 2003) |  | A (Lyu et al. 2023) |  |
| 691 | C_5_H_10_O | 1-Penten-3-ol | 616-25-1 | M, R, S | 90 (6) | 683 | 48 | > | 3 | > |  | (Zhang et al. 2008) |  | R (Lyu et al. 2023) |  |
|  |  | *Furans* |  |  |  |  |  |  |  |  |  |  |  |  |  |
| 703 | C_4_H_6_O_2_ | 2-Ethylfuran | 3208-16-0 | M, R | 79 (4) | 703 | 33 | ApN | ApN | > |  |  | (Sabik et al. 2010) |  |  |

LRI = linear retention index, MF = molecular formula, ID = identification methods, SM = spectral match, given as mean (standard deviation), DF = detection frequency, *Ap*I = ALB-infested, *Ap*N = neighboring to ALB-infestation, *Ap*C = control.

^a^ Identification methods: M = comparison of the mass spectrum with those contained in NIST20 Library, R = comparison of calculated LRI with those contained in NIST20 Library, S = Confirmation with analytical standards

^b^ For inter-tree comparison, ratio was calculated from component areas averaged over period 3 (after *Ap*I has been exposed to ALB), and for intra-tree comparison, ratio was calculated from component areas averaged over period 1–2 (N = 20) and 3 (N = 63), respectively. Only component areas of compounds that were detected in at least 20% of measurements per sample type and period were used for calculation. Ratios of compounds are marked with ‘*Ap*N‘ if they were exclusively detected at *Ap*N, with ‘ALB’ if the respective compound appeared *de novo* in period 3, and with ‘>’ if the compound appeared in less than 20 % of measurements in periods 1–2.

**Table S 3** Stress-induced plant volatiles emitted at the trunks of both Norway maples (*Acer platanoides*) inside the quarantine room, detected in ≥ 50 % of the measurements within the first 24 weeks after ALB exposure to *Ap*I. References comprise VOCs emitted from different parts of *Acer* (spp.) and with relevance to *Sclerodermus* (spp.), showing binding affinities (BA) to odorant-binding proteins in antennae

| LRI  exp. | MF | Compound name | CAS | ID ^a^ | NIST20 | | DF (%) | | Emission rate ratio ^b^ | | | | References | | |
| --- | --- | --- | --- | --- | --- | --- | --- | --- | --- | --- | --- | --- | --- | --- | --- |
|  |  |  |  |  |  |  |  |  | Inter-tree | | Intra-tree pre/post ALB | | *Acer* (spp.) | | Semiochemicals |
|  |  |  |  |  | SM | LRI  lit. | *Ap*N  (N = 63) | *Ap*I  (N = 61) | *Ap*N/ *Ap*C | *Ap*I/ *Ap*C | *Ap*N | *Ap*I | Canopy | Syrup | *Sclerodermus*  (spp.) |
|  |  | *Aldehydes* |  |  |  |  |  |  |  |  |  |  |  |  |  |
| 655 | C_5_H_10_O | 3-Methylbutanal | 590-86-3 | M, R, S | 91 (5) | 652 | 94 | 75 | 4 | 3 | 2 | 2 |  |  |  |
| 967 | C_7_H_6_O | Benzaldehyde | 100-52-7 | M, R, S | 95 (4) | 962 | 95 | 100 | 5 | 3 | 3 | 4 | (Zhang et al. 2008) | (Sabik et al. 2010) | BA (Huang et al. 2023) |
|  |  | *Alcohols* |  |  |  |  |  |  |  |  |  |  |  |  |  |
| 1035 | C_8_H_18_O | 2-Ethyl-1-hexanol | 104-76-7 | M, R, S | 92 (4) | 1030 | 94 | 95 | 4 | 4 | 2 | 2 | (Li et al. 2003; Zhang et al. 2008; Wang et al. 2016) | (Sabik et al. 2010) |  |
| 1045 | C_7_H_8_O | Benzyl alcohol | 3856-25-5 | M, R, S | 94 (6) | 1036 | 63 | 75 | 3 | 3 | 3 | 4 |  |  |  |
|  |  | *Hydrocarbons* |  |  |  |  |  |  |  |  |  |  |  |  |  |
| 894 | C_8_H_8_ | Styrene | 100-42-5 | M, R, S | 88 (6) | 893 | 62 | 61 | 4 | 3 | 3 | > |  |  | BA (Huang et al. 2023) |
| 957 | C_9_H_12_ | Propylbenzene | 103-65-1 | M, R | 88 (4) | 953 | 54 | 67 | > | > | 2 | 2 |  |  |  |

LRI = linear retention index, MF = molecular formula, ID = identification methods, SM = spectral match, given as mean (standard deviation), DF = detection frequency, *Ap*I = ALB-infested, *Ap*N = neighboring to ALB-infestation, *Ap*C = control.

^a^ Identification methods: M = comparison of the mass spectrum with those contained in NIST20 Library, R = comparison of calculated LRI with those contained in NIST20 Library, S = Confirmation with analytical standards

^b^ For inter-tree comparison, ratio was calculated from component areas averaged over period 3 (after *Ap*I has been exposed to ALB), and for intra-tree comparison, ratio was calculated from component areas averaged over period 1–2 (N = 20) and 3 (N = 61–63), respectively. Only component areas of compounds that were detected in at least 20% of measurements per sample type and period were used for calculation. Ratios are marked with ‘>’ if they appeared in less than 20 % of measurements within period 1–2.

# REFERENCES

Huang G, Liu Z, Gu S, et al (2023) Identification and functional analysis of odorant-binding proteins of the parasitoid wasp Scleroderma guani reveal a chemosensory synergistic evolution with the host Monochamus alternatus. Int J Biol Macromol 249. https://doi.org/10.1016/j.ijbiomac.2023.126088

Keena MA (2006) Effects of Temperature on *Anoplophora glabripennis* (Coleoptera: Cerambycidae) Adult Survival, Reproduction, and Egg Hatch. Environ Entomol 35:912–921. https://doi.org/10.1603/0046-225X-35.4.912

Keena MA, Moore PM (2010) Effects of Temperature on *Anoplophora glabripennis* (Coleoptera: Cerambycidae) Larvae and Pupae. Environ Entomol 39:1323–1335. https://doi.org/10.1603/EN09369

Li J, Jin Y, Luo Y, et al (2003) Leaf volatiles from host tree Acer negundo: diurnal rhythm and behavior responses of Anoplophora glabripennis to volatiles in field. Acta Bot Sin 45:177–182

Lyu F, Hai X, Wang Z (2023) A Review of the Host Plant Location and Recognition Mechanisms of Asian Longhorn Beetle. Insects 14:292. https://doi.org/10.3390/insects14030292

Makarow R, Schäfer S, Kaul P (2020) Identification of Anoplophora glabripennis (Moschulsky) by its emitted specific volatile organic compounds. Sci Rep 10:5194. https://doi.org/10.1038/s41598-020-61897-0

Sabik H, Fortin J, Martin N (2010) Identification of volatile compounds in maple syrup using headspace solid-phase microextraction with gas chromatography-mass spectrometry. In: Chromatography: Types, Techniques and Methods. Nova Science Publishers, Inc., pp 417–427

Wang Q, Liu H, Wang B, et al (2016) Component analysis of volatile organic compounds from branches and leaves in seven Acer species. Zhejiang Nonglin Daxue Xuebao 33:524–530

Xu T, Hansen L, Cha DH, et al (2020) Identification of a female-produced pheromone in a destructive invasive species: Asian longhorn beetle, Anoplophora glabripennis. J Pest Sci 93:1321–1332. https://doi.org/10.1007/s10340-020-01229-3

Zhang F, Jin Y, Chen H, Wu X (2008) Selectivity mechanism of Anoplophora glabripennis on four different species of maples. Frontiers of Biology in China 3:78–84. https://doi.org/10.1007/s11515-008-0006-1
